# Supplementary material for: Learning as filtering: Implications for spike-based plasticity
Source: PLoS Comput Biol. 2022 Feb 23;18(2):e1009721. doi: 10.1371/journal.pcbi.1009721 (PMC8865661; doi:10.1371/journal.pcbi.1009721)
Supplement: S1 Text — S1 Fig. The Sampling Synaptic Filters have similar MSEs to their deterministic counterparts. S2 Fig. The first and second moments of the Synaptic Filter match the corresponding moment of the exact filtering distribution. S3 Fig. Optimisation of the learning rate for the gradient rule. S4 Fig. The dynamics of the variables of the Synaptic Filter during the STDP protocol. S5 Fig. The dynamics of the variables of the Synaptic Filter during the heterosynaptic protocol. (ZIP) [file pcbi.1009721.s001.zip › S1_Text.pdf]

Supplementary Information of the paper "Learning as filtering: implications for spike-based plasticity"

Jannes Jegminat, Simone Carlo Surace and Jean-Pascal Pfister

The Supplementary Information addresses three main questions. Section A asks whether the sampling hypothesis is compatible with good MSE performance (low MSE values). In Section B, we analyse whether the Synaptic Filter is a faithful solution filtering problem. Thirdly, in Section C, we answer how the update equations of the Synaptic Filter (Equations (1) and (2) in the Main Text, here Equations (S36) and (S37)) are derived.

Additional short sections provide additional explanations for the results in the Main Text. Section D details how the performance was evaluated for the gradient rule and Section E shows how the variables of the Synaptic Filter evolve during the plasticity protocols. In the final Section, we discuss when weight correlations are negative in the Synaptic Filter with more than two dimensions.

Note that unlike in the Main Text, the performance simulations in Section A and B are reported in terms of dimension-independent factor of the determinism: β_0. Specifically, $\beta = \beta_0 d^{-1/2}$ with dimension d.

A The sampling hypothesis is compatible with low MSE

The sampling hypothesis states that EPSPs are samples from the filtering distribution $p(w_t|\mathcal{D}_t)$ [1]. However, the Synaptic Filter, as derived before, does not use weight sampling. We wondered whether the sampling hypothesis could be used to inspire a version of the Synaptic Filter that maintains good filtering performance while at the same time computing the expected firing rate through EPSP samples.

Based on two heuristic modifications of the update equations of the Synaptic Filter Equations (S36) and (S37), we derive the Sampling Synaptic Filter. The first modification is that the expected firing rate γ_t is replaced with the sampling based firing rate $\gamma_t^{\mathrm{s}} := g(u_t^{\mathrm{s}})$. The sampling based membrane potential u_t^{s} is directly motivated by the sampling hypothesis, i.e., it is a low-pass filtered sum of spike-triggered samples from all synapses. The second modification aims at slowing down learning to the sampling time scale τ_{s}. This modification is motivated by the empirical observation that good filtering performance requires the sampling based firing rate γ_t^{s} to change on the same time scale as other spike-related terms in the update Equations (S36) and (S37). In the following, both modifications are discussed in more detail.

The sampling based firing rate $\gamma_t^{\mathrm{s}} := g(u_t^{\mathrm{s}})$ is computed on the basis of a sampling based membrane potential u_t^{s}, which is modelled as leaky-integrator of spike-triggered EPSPs w_t^{s}:

$$u_t^{\mathrm{s}} = ((w^{\mathrm{s}})^\top x * \epsilon)_t. \tag{S1}$$

The sum $(w^{\mathrm{s}})^\top x$ takes a value different from zero only when a spike arrives at synapse $0 < i < d$ and a sample from the marginal filtering distribution is drawn:

$$w^{\mathrm{s}}_{t,i} \sim \mathcal{N}(\mu_{t,i}, \Sigma_{t,ii}). \tag{S2}$$

Since no two spikes occur in the same moment, the Sampling Synaptic Filter does not include correlations between weights. The bias is treated as a special case because it does not receive spikes and, thus, the idea of spike-triggered sampling cannot be applied. We make the assumption that Sampling Synaptic Filter includes the bias in the sampling based firing rate γ^{s}_t in the same way as the Synaptic Filter, i.e., we adopt the following convention for the first component of the sum:

$$(w^{\mathrm{s}}_0 x_0 * \epsilon)_t \equiv \mu_{t,0} + \tfrac{1}{2}\beta\sigma^2_{t,00}. \tag{S3}$$

With Equation (S3) and in the case of $d = 1$, the expected firing rate of the Synaptic Filter and sampling based firing rate in the Sampling Synaptic Filter are identical: $\gamma^{\mathrm{s}}_t = \gamma_t$.

The second modification aims at slowing down learning in order to suppress fast feedback dynamics between weights and expected firing rate. Without this suppression, filtering performance deteriorates because the first modification prevents the fast feedback from working properly. Consider how the fast feedback works in the case of the Synaptic Filter. A large expected firing rate γ can reduce its own value indirectly by reducing the mean and variance values of all weights with active inputs through the update Equations (S36) and (S37). This reduction can be faster than the membrane time scale τ_{m}. In the Sampling Synaptic Filter, this fast feedback mechanism does not work anymore. The sampling based firing rate γ^{s}_t depends on samples of past filtering distributions and cannot influence the value of these samples through the update Equations (S36) and (S37). The sampling based firing rate γ^{s}_t, responds to changes in the filtering distribution with a delay of τ_{m}. Thus, the weight updates can overshoot which leads to poor performance.

One way to address the broken fast feedback is by introducing a delay in the updates in Equations (S36) and (S37) such that the delayed response of γ^{s}_t to changes in the filtering distribution becomes irrelevant. Specifically, we consider spike related quantities as fast and replace them by their low-pass filtered version. With the exponential kernel α with *sampling time scale* τ_{s}, the update Equations (S36) and (S37) of the Sampling Synaptic Filter are defined as follows:

$$\dot{\mu}_t = \beta\Sigma_t\left((\alpha * x^\epsilon y)_t - (\alpha * x^\epsilon \gamma^{\mathrm{s}})_t\right) - \tau_{\mathrm{ou}}^{-1}(\mu_t - \mu_{\mathrm{ou}}) \tag{S4}$$

$$\dot{\Sigma}_t = -\beta^2\Sigma_t(\alpha * x^\epsilon (x^\epsilon)^\top \gamma^{\mathrm{s}})_t\Sigma_t - 2\tau_{\mathrm{ou}}^{-1}(\Sigma_t - \Sigma_{\mathrm{ou}}). \tag{S5}$$

In all simulations, we chose $\tau_{\mathrm{s}} = 4\tau_{\mathrm{m}} = 100$ms. Additionally, we test the Diagonal Sampling Synaptic Filter which is updated according to Equations (S4) and (S5) with the only difference that a diagonal covariance matrix is enforced. While both versions of the Sampling Synaptic Filter compute the sampling membrane based on the marginal filtering distribution, i.e., without considering correlations between samples, the Sampling Synaptic Filter includes the off-diagonal elements of the covariance matrix during learning while the Diagonal Sampling Synaptic Filter uses a diagonal covariance matrix.

Because the model of the membrane potential differs between the sampling filters and their deterministic counterparts, Equations (S4) and (S5) do not converge to Equations (S36) and (S37) in a non-trivial limiting case. Furthermore, the history of the weight samples w^{s}_t must be included in the data $\mathcal{D}^{\mathrm{s}}_t := (w^{\mathrm{s}}_{0:t-\mathrm{d}t}, \mathcal{D}_t)$. The key advantage of the Sampling Synaptic Filter over the Synaptic Filter is that it does not require the

instantaneous evaluation of the expected firing rate γ_t but instead uses a biologically more plausible expected firing rate γ_t^{s} inspired by the sampling hypothesis.

In the following, the MSE of the Synaptic Filter, the Sampling Synaptic Filter, the Diagonal Synaptic Filter and the Diagonal Sampling Synaptic Filter are evaluated for a range of values for the determinism parameter β_0. We used 100 simulations[1] with an OU time scale of $\tau_{\mathrm{ou}} = 100$ s and duration $T_{\mathrm{sim}} = 10\tau_{\mathrm{ou}}$. A burn in of 200 s was used to reduce the dependence on the initial conditions, which was a random sample from the equilibrium distribution of the prior.

Taking into account the sampling hypothesis does not substantially impair performance, i.e., the MSE does increase, as shown in in Fig S1. The sampling filters behave similarly to their deterministic counterparts, including a small performance gain from using the covariance matrix (red lines) in the update equations. Still, the Synaptic Filter (red solid line) remains the best model overall.

The ultimate goal of a neuron is to make prediction. From a Bayesian perspective, the posterior predictive distribution is optimal for making predictions. Bayesian regression takes parameter uncertainty into account, thus being more robust against overfitting. However, computing the posterior predictive distribution is challenging because it involves the posterior expectation of the prediction function. For a Poisson neuron, the posterior predictive is the Poisson process of the generative model with the difference that the expected firing rate γ_t is the rate parameter. We hypothesise that neurons could approximate γ_t with γ_t^{s}, thereby taking advantage of parameter uncertainty during predictions.

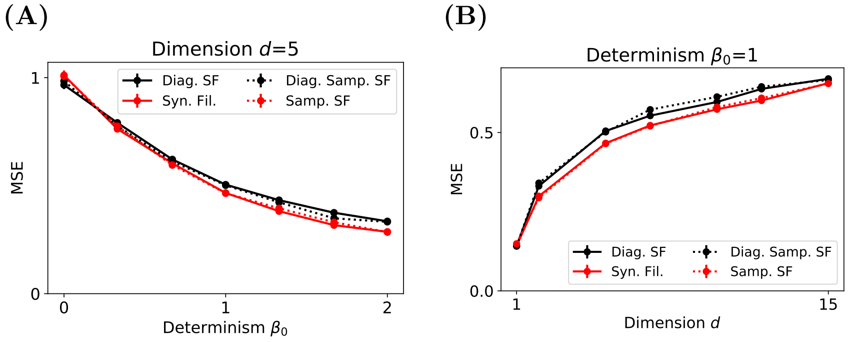

Fig S1. The Sampling Synaptic Filters have similar MSEs to their deterministic counterparts for all values of the dimension d and determinism β_0. **(A)** The MSE of Synaptic Filter (red solid), Sampling Synaptic Filter (red dashed) and their diagonal counterparts (black) decrease as the determinism β_0 increases. **(B)** For all filtering models, the MSE increases as a function of dimension. Again, the diagonal variants (black) perform worse. Dots and errors denote the mean and SEM from 100 simulations. The simulated time per run was $10\tau_{ou} = 1000$ s.

B The Synaptic Filter solves the filtering problem

B.1 The normalised moments of the Synaptic Filter correspond to the moments of the exact filtering distribution

The Synaptic Filter is an approximated solution to the filtering problem. Thus, we need to determine the level of accuracy of the approximation. Specifically, we check if the

[1] When a discretisation in the firing rate occurred, i.e., $g(u_t)\mathrm{d}t > 1$, we corrected it by enforcing a value of 1. Less than 10^{-4} of the time steps needed a correction, which we regarded within error tolerance. The time step was $\mathrm{d}t = 1$ ms.

moments of the Synaptic Filter are consistent with the corresponding moments of the exact filtering distribution. We test a range of values for the determinism parameter β with fixed dimension $d = 5$, and a range of dimensions d for a fixed value of the determinism β. To compare the moments of the Synaptic Filter $q_{\theta_t}(w_t)$ with the moments of the exact filtering distribution $p(w_t|\mathcal{D}_t)$, we compute the normalised estimators $z^{(1)}$ and $z^{(2)}$ of the first and second moment respectively. If the moments of q converge to the first two moments of the exact distribution, the normalised estimators converge $z^{(1)} \to 0$ and $z^{(2)} \to 1$ (see Section B.2). We use these conditions to measure the quality of the Synaptic Filters. Additionally, we obtain an approximation to the exact filtering distribution $p(w_t|\mathcal{D}_t)$ with a particle filter (see Section B.4). The particle filter converges to the exact solution of the filtering problem in the limit of a large number of particles.

The results in Fig S2A-D show that, for a range of dimensions d and values of the determinism β_0, the normalised estimators of the Synaptic Filter (SF) confirm consistency between the approximated and the exact filtering distribution, i.e. $z^{(1)}_{\mathrm{SF}} \approx 0$ and $z^{(2)}_{\mathrm{SF}} \approx 1$. The Synaptic Filter (red solid line) and the Diagonal Synaptic Filter (black solid line) show similar performance at $d = 1$ (Fig S2B and S2D) because in this case the covariance matrix is a scalar and, hence, both models are identical. However, at higher dimensions the Diagonal Synaptic Filter exhibits a small deviation from $z^{(1)} = 0$ and a strong positive deviation from $z^{(2)} = 1$. For the later, the deviation grows linearly with the determinism β_0, as shown in Fig S2C.

The reason is that due to its omission of correlations, the Diagonal Synaptic Filter underestimates the overall weight uncertainty, and hence overestimates the overall precision and $z^{(2)}$, which is proportional to the precision matrix, i.e., the inverse of the covariance matrix. Correlations arise from the likelihood term in the update Equation (S37), which is proportional to β_0. This explains the scaling of the deviation with β_0. The superior performance of the Synaptic Filter shows that the off-diagonal elements of the covariance matrix are important to obtain a good approximation to the filtering distribution.

The estimators computed based on the particle filter (gray) are generally consistent with the exact distribution and with the Synaptic Filter, i.e., they satisfy $z^{(1)}_{\mathrm{PF}} \to 0$ and $z^{(2)}_{\mathrm{PF}} \to 1$. This was expected since particle filters are asymptotically exact in the limit of infinitely many particles. The systematic deviation $z^{(2)}_{\mathrm{PF}} > 0$ in (Fig S2D) arises because particle filters suffer from the curse of dimensionality, which leads to underestimates of the covariance in higher dimensions.

The Sampling Synaptic Filter (red dashed line) and the Diagonal Sampling Synaptic Filter (black dashed line) estimate the second moment $z^{(2)}$ with similar accuracy as their counterparts without sampling (solid lines), as shown in Fig S2C and S2D. The Sampling Synaptic Filter performs well, i.e., $z^{(2)}_{\mathrm{SSF}} \to 1$, while the Diagonal Sampling Synaptic Filter deviates strongly from $z^{(2)} = 1$. Both sampling filters perform well in terms of the first normalised moment, shown in Fig S2A and S2B. The fact that both sampling filters estimate the moments of the exact filtering distribution with comparable accuracy as their deterministic counterparts implies that the explicit inclusion of the sampling hypothesis in the updates does not impair filtering performance. For this conclusion to hold, we had to assume a sampling time scale τ_{s} that was many orders of magnitude smaller than the time scale of the weight evolution τ_{ou} (see Main Text).

The analysis of the first and second normalised moment estimators, $z^{(1)}$ and $z^{(2)}$, shows that the mean and covariance computed by the Synaptic Filter correspond closely to the mean and covariance of the exact filtering distribution. A particle filter solution to the filtering problem confirms this. The fact that the Diagonal Synaptic Filter and the Diagonal Sampling Synaptic Filter perform poorly shows that off-diagonal elements

in the covariance matrix must be included to match the moments of the exact filtering distribution.

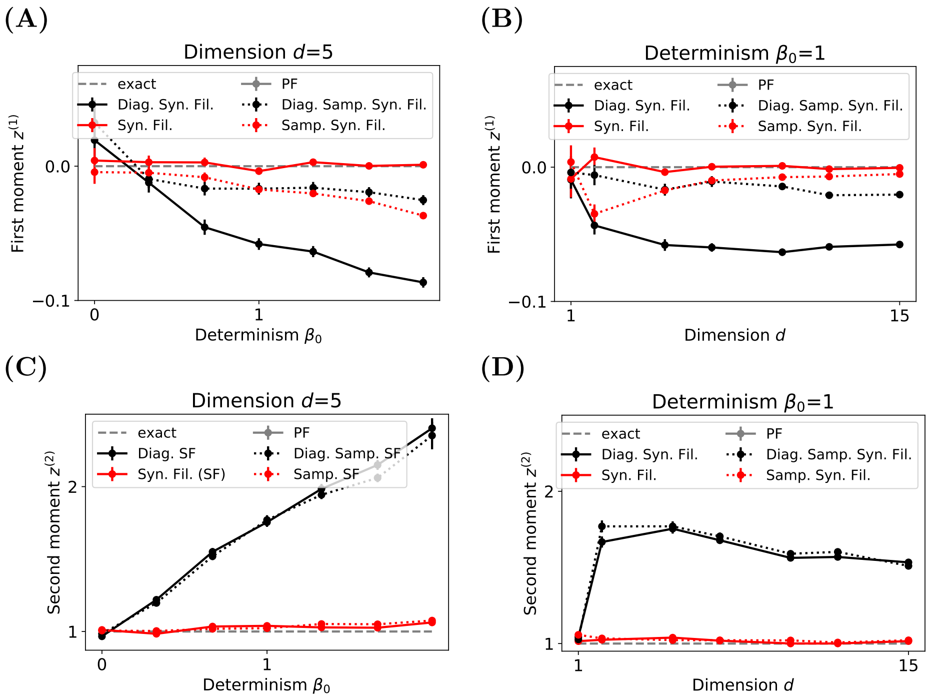

Fig S2. The first and second moments of the Synaptic Filter (SF, solid red line) match the corresponding moment of the exact filtering distribution, i.e. the normalised moments of the Synaptic Filter satisfy $z^{(1)}_{\mathrm{SF}} \approx 0$ and $z^{(2)}_{\mathrm{SF}} \approx 1$. **(A)** For $d = 5$ and a $0 \leq \beta_0 \leq 2$, the first normalised moment $z^{(1)}$ of the Synaptic Filter and the particle filter (PF, gray line) are close to 0 while the Sampling Synaptic Filter (SSF, solid black), Diagonal Synaptic Filter (DSF, dashed red line), and Diagonal Sampling Synaptic Filter (DSSF, dashed black line) deviate from 0. At $\beta_0 = 0$, all filtering distributions resemble the prior. As the determinism β_0 increases, the deviation increases as well. **(B)** For $\beta_0 = 1$ and $1 \leq d \leq 15$, the first normalised estimator $z^{(1)}$ of all models except for the Diagonal Synaptic Filter are close. The dimension d corresponds to the number of presynaptic inputs plus one (for the bias) so when $d = 1$, the Synaptic Filter and Sampling Synaptic Filter (red lines) are equivalent to their diagonalised (black). However, the diagonalised versions perform worse for $d > 2$. **(C)** The second normalised estimator $z^{(2)}$ of the Synaptic Filter, the Sampling Synaptic Filter and the particle filter are close to 1 while the diagonalised versions (black) overestimate $z^{(2)}$. The deviation is linear in β_0. **(D)** As before, the $z^{(2)}$ is close to one for Synaptic Filter and Sampling Synaptic Filter and deviates for the diagonal models. The particle filter is consistent with $z^{(2)} = 1$ at low dimensions but deviates systematically for increasing d. Dots and error bars denote the mean and SEM from 100 simulations. The simulation details are given in Section A.

B.2 Time averaged normalised moment estimators

The naive approach to quantify whether the moments of a proposal distribution, i.e., an approximation to the exact filtering distribution, match the moments of the exact filtering distribution, is computing the moments of both. However, this requires the potentially expensive computation of the moments of the exact filtering distribution. To avoid this problem, we use an implicit comparison via the normalised moments. In particular, we calculate the first two normalised moments $z^{(1)}$ and $z^{(2)}$, which form the basis of the analysis in Section B.1.

Consider a proposal distribution $q_\theta(w_t)$ with mean μ_t and covariance Σ_t. The moments of the proposal can be compared to the respective moments $\mu^\star_t$ and $\Sigma^\star_t$ of the exact filtering distribution $p(w_t|\mathcal{D}_t)$ by averaging over many realisations of the

generative model, i.e., over the history of the hidden weight $w_{0:t}$ and data generated $\mathcal{D}_t$. To see this, we rewrite the distribution over the realisations of the generative model using the definition of conditional probabilities:

$$p(w_{0:t}, \mathcal{D}_t) = p(w_t, w_{0:t-\mathrm{d}t}|\mathcal{D}_t)p(\mathcal{D}_t). \tag{S6}$$

Now, we define the time dependent, normalised moments $z_t^{(1)}$ and $z_t^{(2)}$ as a function of the hidden weight w_t and the moments of the proposal:

$$z_t^{(1)} := d^{-1} \sum_{i=1}^{d} (\Sigma_t^{-\frac{1}{2}}(w_t - \mu_t))_i \tag{S7}$$

$$z_t^{(2)} := d^{-1}(w_t - \mu_t)^\top \Sigma_t^{-1}(w_t - \mu_t). \tag{S8}$$

Since Equations (S7) and (S8) do not depend on $w_{0:t-\mathrm{d}t}$, the expectation with respect to realisations of the generative model (Equation (S6)) is equivalent to the expectation with respect to the posterior and the data, i.e. $p(w_t|\mathcal{D}_t)p(\mathcal{D}_t)$:

$$\mathbb{E}[z_t^{(1)}|w_{0:t}, \mathcal{D}_t] = d^{-1} \sum_{i=1}^{d} \mathbb{E}[(\Sigma_t^{-\frac{1}{2}}(\mu_t^\star - \mu_t))_i|\mathcal{D}_t] \tag{S9}$$

$$\mathbb{E}[z_t^{(2)}|w_{0:t}, \mathcal{D}_t] = d^{-1}\mathrm{Tr}\left(\mathbb{E}[\Sigma_t^{-1}(\mu_t^\star - \mu_t)(\mu_t^\star - \mu_t)^\top + \Sigma_t^{-1}\Sigma_t^\star|\mathcal{D}_t]\right), \tag{S10}$$

where we evaluated w_t using the mean and covariance of the exact posterior. To obtain Equation (S10), we further used that the trace operator leaves a scalar quantity invariant and permits cyclic permutation. Note that the moments of the exact posterior and the proposal dependent on the history of the data $\mathcal{D}_t$, which is why the expectation on the right-hand side of Equations (S9) and (S10) remains. However, in the special case that the proposal is equivalent to the exact posterior, we have $\mu_t^\star = \mu_t$ and $\Sigma_t^{-1}\Sigma_t^\star = \mathbb{1}$, and consequently:

$$\mathbb{E}[z_t^{(1)}|w_{0:t}, \mathcal{D}_t] = 0 \tag{S11}$$

$$\mathbb{E}[z_t^{(2)}|w_{0:t}, \mathcal{D}_t] = 1. \tag{S12}$$

Thus, Equations (S9) and (S10) can be compared to Equations (S11) and (S12) to test whether the moments of a proposal distribution match the moments of the exact posterior without explicitly calculating the latter.

The quantities shown in Fig S2 are time averaged estimates of Equations (S9) and (S10) computed from 100 realisations of the generative model. The time average runs over 10 τ_{ou} time constants. The average over time is justified because the generative model is stationary, i.e., there is no explicit dependence on time. Indexing realisations by k, we define the normalised moments by:

$$z^{(1)} := \tfrac{1}{100} \sum_{k=1}^{100} \langle z_{t,k}^{(1)} \rangle_t \tag{S13}$$

$$z^{(2)} := \tfrac{1}{100} \sum_{k=1}^{100} \langle z_{t,k}^{(2)} \rangle_t, \tag{S14}$$

where $\langle \cdot \rangle_t$ denotes the time averaging.

B.3 Simulation details for Fig 2A in the Main Text

In the Main Text in Fig 2A, the MSE of the gradient rule is shown as a function of the learning rate along with MSE of the Synaptic Filter. These results were obtained along

side with the simulation of the Sampling Synaptic Filter (Section A) and the evaluation of the the normalised moments (Section B.1). The membrane time constant was $\tau_{\mathrm{m}} = 25$ ms, the base firing rate $g_0 = 1$ Hz, the input firing rate $\nu_0 = 40$ Hz and the time scale of the OU-process was $\tau_{\mathrm{ou}} = 100$ s. In contrast to the other performance simulations in the Main Text, a bias was included (by setting the spike train of the first input neuron to unity) and we used 100 simulations with 10 epochs each. For each simulation, a burn-in of two epochs was used. Learning rate values for the gradient rule were taken from the interval $10^{-3}, \ldots, 10^{1}$ but the highest values lead to divergences as one would expect and were discarded from the plot.

B.4 Particle filter

To compute an asymptotically correct approximation to the posterior distribution (without the approximation of the Assumed Density Filter) we use a particle filter with $L = 2^{13}$ particles (see gray lines in Fig S2). The update of the position $v^{(l)}$ and importance weight $a^{(l)}$ of the lth particle are given by:

$$\mathrm{d}v_t^{(l)} = \frac{\mu_{\mathrm{ou}} - v_t^{(l)}}{\tau_{\mathrm{ou}}}\mathrm{d}t + \sqrt{2\sigma_{\mathrm{ou}}^2 \tau_{\mathrm{ou}}^{-1}}\mathbb{1}\mathrm{d}V_t \tag{S15}$$

$$\mathrm{d}a_t^{(l)} = a_t^{(l)} \left(\frac{g(\beta(v_t^{(l)})^\top x_t^{\epsilon})}{\langle g_t \rangle} - 1 \right) (y_t - \langle g_t \rangle)\mathrm{d}t, \tag{S16}$$

where the average $\langle g_t \rangle = \sum_{l=1}^{L} a_t^{(l)} g(\beta(v_t^{(l)})^\top x_t^{\epsilon})$ is computed based on the particle weights and their location. When the effective particle number $N_t = (\sum_{l=1}^{L}(a_t^{(l)})^2)^{-1} < \frac{3}{4}L$, we resample the particle location and set all weights to $1/L$.

Discretisation errors can lead to negative particle weights, which we corrected by setting them to 0 and renormalising all particle weights afterwards. High values of β_0 caused the highest frequency of discretisation errors. For $\beta_0 = 2$ and $\beta_0 = 1.67$ negative particle weights occurred in 0.5% and 0.1% of the time steps. We tolerated these errors because the values of the negative importance weights were always close to zero compared to the remaining ones and thus did not substantially affect the accuracy of the particle filter.

The initial positions of the particles, indexed with l, were drawn from the prior as well: $v_{t=0}^{(l)} \sim \mathcal{N}(\mu_{\mathrm{ou}}, \Sigma_{\mathrm{ou}})$. After initialisation, a burn-in period of τ_{ou} was simulated. Additional details about particle filtering are found in the literature, e.g. [2].

C Derivation of the Synaptic Filter

The derivation of the Synaptic Filter extends the work of Pfister et al. [3] to multidimensional hidden variables and a more complex observation process. The starting point of the derivation is the general framework of filtering with point observations and hidden diffusion dynamics. Then, the assumed density filter is introduced as a strategy to solve the filtering problem. The next two sections specify the generative model of the Synaptic Filter and show how it yields the update equations used in the Main Text.

C.1 Filtering with point process observations

Given the continuous time spiking observations $y_t = \sum_f \delta(t - t^{(f)})$ from hidden weights $w_t \in \mathbb{R}^d$, where $t^{(f)}$ are the spiking times, our goal is to derive the update equations for

the parameters θ_t of the proposal distribution $q_{\theta_t}(w_t)$ of an assumed density filter. The generative model is specified in terms of a prior transition probability and point emission process. For the transition, we consider diffusion processes of the form:

$$\mathrm{d}w_t = a(w_t)\mathrm{d}t + b(w_t)\mathrm{d}V_t, \tag{S17}$$

where V_t is a d-dimensional Wiener process and $a \in \mathbb{R}^d \to \mathbb{R}^d$ and $b \in \mathbb{R}^d \to \mathbb{R}^{d\times d}$ are deterministic functions. The observation $\mathrm{d}N_t \in \{0, 1\}$ indicates whether a spike is present in the infinitesimal interval $\mathrm{d}t$:

$$\mathrm{d}N_t \sim \mathrm{Poisson}(g_t(w_t)\mathrm{d}t), \tag{S18}$$

where the (deterministic) gain function $g_t(w_t)$ relates the hidden weights to observations and $y_t = \mathrm{d}N_t/\mathrm{d}t$.

This non-linear filtering problem has a general solution given by a Kushner-Stratonovic type of equation for point processes [4-6]. Using the Laplacian $\mathcal{L}(\cdot) = a^\top\nabla(\cdot) + \frac{1}{2}\mathrm{Tr}(bb^\top\nabla\nabla^\top(\cdot))$ all moments ϕ_t of the posterior obey the following formal solution:

$$\mathrm{d}\langle\phi_t\rangle = \langle\mathcal{L}(\phi_t)\rangle\mathrm{d}t + \frac{\mathrm{cov}(\phi_t, g_t)}{\gamma_t}\mathrm{d}\delta_t, \tag{S19}$$

where the expectation $\langle\cdot\rangle$ is taken with respect to the filtering distribution and we introduced the error signal $\mathrm{d}\delta_t = \mathrm{d}N_t - \gamma_t\mathrm{d}t$ based on the expected firing rate $\gamma_t := \langle g_t\rangle$.

Generally, Equation (S19) is intractable due to the closure problem, i.e., the evolution of n-th moment depends on the $n+1$-th moment. For instance, for the choice $g_t \equiv w_t \in \mathbb{R}$, the evolution of the first moment ($\phi_t(w_t) \equiv w_t$) depends on the variance $\mathrm{cov}(w_t^2)$. However, when Equation (S19) is used to compute the evolution of the variance ($\phi_t(w_t) = w_t^2$), third order terms appear: $\mathrm{cov}(w_t^2, w_t)$.

C.2 Assumed density filter with Gaussian proposal

One strategy to apply Equation (S19) is assumed density filtering (e.g. [7]). The central idea is to replace the exact filtering distribution $p(w_t|\mathcal{D}_t)$ with a more tractable proposal distribution $q_{\theta_t}(w_t)$ with parameters $\theta_t \in \mathcal{S} \subset \mathcal{R}^r$. The fact that the proposal distribution belongs to a parametric family, limits its degrees of freedom. Thus, (in the absence of degeneracy) the number of parameters r determines how many moments have to be computed in order to fully specify the evolution of the proposal distribution. The closure problem can be avoided.

The Synaptic Filter and the Diagonal Synaptic Filter are assumed density filters with a Gaussian proposal distribution:

$$q_{\theta_t}(w_t) := \mathcal{N}(w_t; \mu_t, \Sigma_t). \tag{S20}$$

In the case of the Diagonal Synaptic Filter Σ_t contains only diagonal elements. The general notation Σ_t (with or without off-diagonal elements) allows for the simultaneous derivation of both filters. To relate the parameters of the proposal $\theta_t = (\mu_t, \Sigma_t)$, we follow a simple moment-matching strategy, i.e., we use Equation (S19) to directly compute the updates for θ_t. In the case of Gaussian distributions, moment matching is optimal in the sense that it minimizes the Kullback-Leibler divergence: $\mathcal{D}_{KL}(p(w_t|\mathcal{D}_t)|q_{\theta_t}(w_t))$. However, the question of how to optimally project the exact filtering distribution $p(w_t|\mathcal{D}_t)$ onto the proposal is an active research area in information geometry [8].

To compute the evolution of θ_t, we specify Equation (S19) for the first two central moments μ_t and Σ_t ([9], Equations (101) and (102)):

$$\mathrm{d}\mu_t = \langle a_t \rangle \mathrm{d}t + \frac{\mathrm{cov}(w_t, g_t)}{\gamma_t} \mathrm{d}\delta_t \tag{S21}$$

$$\begin{aligned}\mathrm{d}\Sigma_t = & (\mathrm{cov}(a_t, w_t^\top) + \mathrm{cov}(w_t, a_t^\top) + \langle b_t b_t^\top \rangle)\mathrm{d}t \\ & + \frac{\mathrm{cov}(w_t w_t^\top, g_t) - \mu_t \mathrm{cov}(w_t^\top, g_t) - \mathrm{cov}(w_t, g_t)\mu_t^\top}{\gamma_t} \mathrm{d}\delta_t \\ & - \frac{\mathrm{cov}(w_t, g_t)\mathrm{cov}(w_t^\top, g_t)}{\gamma_t^2} \mathrm{d}N_t.\end{aligned} \tag{S22}$$

Expectations $\langle \cdot \rangle$ are now evaluated with respect to the proposal density $q_{\theta_t}(w_t)$ rather than with respect to the exact filtering distribution $p(w_t|\mathcal{D}_t)$. For the Diagonal Synaptic Filter, one considers only the updates of the diagonal elements in Equation (S22). In general, the updates of the diagonal elements can have complex dependencies on off-diagonal elements, e.g., if we had assumed a non-diagonal matrix b, the term $bb^\top$ would have introduced such mixing of components. However, since b was assumed to be a diagonal matrix and w and a a vectors, no mixing occurs in Equation (S22). This is why in our case the updates of diagonal covariance elements in Diagonal Synaptic Filter and the Synaptic Filter are identical.

C.3 OU-prior and exponential gain function

To evaluate the terms in the moment evolution Equations (S21) and (S22), we must make specific choices for the transition probability, often simply referred to as *prior* and the gain function g_t in the point emission process Equation (S18).

In our work, the emission probability reflects the output spiking of a neuron with membrane potential:

$$u_t = w_t^\top (x * \epsilon)_t \tag{S23}$$

where ϵ denotes the spike response kernel to given input spike trains $x_{t,i} = \sum_f \delta(t - t_i^{(f)})$. When the zero-th weight $w_{t,0}$ acts as bias (Section 2.3, 2.4 and 2.5 in the Main Text), we adopt the convention $(x_0 * \epsilon)_t = 1$. The assumption of an exponential gain function has analytical advantages and corresponds to a neuron close to the onset of a sigmoidal gain function:

$$g_t(w_t) \equiv g_0 \exp(\beta w_t^\top x_t^\epsilon) \equiv g(u_t). \tag{S24}$$

The determinism parameter β can be absorbed in the units of x_t^ϵ and is ommited from the rest of the derivation. In addition, we drop the temporal index, since the right-hand side in Equations (S21) and (S22) is evaluated exclusively at time t. For brevity, we refer to the proposal distribution of the assumed density filter $q_{\theta_t}(w_t)$ as filtering distribution from here on.

For the transition probability of the hidden weights, we use an OU-process with equilibrium values μ_{ou} and $\Sigma_{\mathrm{ou}} = \mathbb{1}\sigma_{\mathrm{ou}}^2$, and relaxation time scale τ_{ou}:

$$a(w) \equiv -\tau_{\mathrm{ou}}^{-1}(w - \mu_{\mathrm{ou}}), \qquad b(w) \equiv \sqrt{\frac{2\sigma_{\mathrm{ou}}^2}{\tau_{\mathrm{ou}}}}\mathbb{1}. \tag{S25}$$

With Equations (S24) and (S25) and the update Equations (S21) and (S22) and the proposal density Equation (S20), the assumed density filter is fully specified. The rest of derivation is dedicated towards the explicit computation of the terms in the update Equations (S21) and (S22).

C.4 Explicit computation of terms in the update equations

C.4.1 Prior

First, the terms related to the transition probability in Equations (S21) and (S22) are evaluated based on Equation (S25):

$$\langle a(w)\rangle = a(\mu) \tag{S26}$$

$$\langle bb^\top\rangle = 2\sigma_{\mathrm{ou}}^2\tau_{\mathrm{ou}}^{-1}\mathbb{1} \tag{S27}$$

$$\begin{aligned}\mathrm{cov}(a, w^\top) &= \langle aw^\top\rangle - \langle a\rangle\langle w^\top\rangle \\ &= -\tau_{\mathrm{ou}}^{-1}(\mu\mu^\top + \Sigma - \mu_{\mathrm{ou}}\mu^\top) + \tau_{\mathrm{ou}}^{-1}(\mu - \mu_{\mathrm{ou}})\mu^\top = -\tau_{\mathrm{ou}}^{-1}\Sigma,\end{aligned} \tag{S28}$$

For the stimulated STDP experiments in the Main Text, we consider a fast time scale τ_{m} for the bias and a slow time scale τ_{ou} for the remaining weights. The update of the covariance element Σ_{0i}, which represents the correlations between the bias and the ith weight, contains both time scales because of the first two terms on the left-hand side of Equation (S22):

$$\mathrm{cov}(a_i, w_0) + \mathrm{cov}(w_i, a_0) = -(\tau_{\mathrm{m}}^{-1} + \tau_{\mathrm{ou}}^{-1})\Sigma_{0i}. \tag{S29}$$

However, since the values of the time scales differ by nine orders of magnitude in the STDP simulations, the contribution of τ_{ou} can be safely ignored.

C.4.2 Observations

Next, we evaluate the terms in Equations (S21) and (S22) that depend on the gain function. We begin by showing that the membrane potential is Gaussian under the statistics of the filtering distribution. Then we evaluate the Gaussian expectation of the gain function. Finally, the expectation of the gain function is used to compute the covariance-terms in Equations (S21) and (S22).

The weighted sums of a Gaussian random variables yields another Gaussian random variable. With the input kernels x^ϵ given and $q_\theta(w) = \mathcal{N}(w; \mu, \Sigma)$, the membrane potential is therefore Gaussian with mean and covariance:

$$\mathbb{E}[u|\theta] = \mu^\top x^\epsilon := \bar{u} \tag{S30}$$

$$\mathrm{Var}[u|\theta] = (x^\epsilon)^\top \Sigma x^\epsilon := \sigma_u^2. \tag{S31}$$

Thus, for any function $g(u)$, the expectation over the d-dimensional filtering distribution can be replaced by the 1-dimensional expectation over the membrane distribution. For the exponential gain function this yields:

$$\mathbb{E}[g|\theta] = g_0 \int e^u \mathcal{N}(u; \bar{u}, \sigma_u^2)\mathrm{d}u = g_0 \exp(\bar{u} + \tfrac{1}{2}\sigma_u^2) \underbrace{\int \mathcal{N}(u; \bar{u} + \sigma_u^2, \sigma_u^2)\mathrm{d}u}_{=1} := \gamma \tag{S32}$$

where the second equality follows from completion of the square in the exponent of the Gaussian. The expected firing rate $\gamma := g_0 \exp(\bar{u} + \frac{1}{2}\sigma_u^2)$ plays a central role in the update equations and for making predictions with the filtering distribution in Bayesian regression.

To compute the covariance terms in Equations (S21) and (S22) we use the fact that the expectation over the filtering distribution commutes with derivatives with respect to x^ϵ:

$$\mathrm{cov}[g, w] := \langle wg\rangle - \langle w\rangle\langle g\rangle = \langle \nabla_x g\rangle - \mu\gamma = \nabla_x \gamma - \mu\gamma = (\mu + \Sigma x^\epsilon)\gamma - \mu\gamma = \Sigma x^\epsilon \gamma \tag{S33}$$

The result $\nabla_x \gamma = (\mu + \Sigma x^\epsilon)\gamma$ can be reused to compute the higher order covariance term in Equation (S22):

$$\begin{aligned}
\mathrm{cov}[g, ww^\top] &:= \langle ww^\top g\rangle - \langle g\rangle\langle ww^\top\rangle \\
&= \nabla_x \nabla_x^\top \gamma - \gamma(\mu\mu^\top + \Sigma) \\
&= \nabla_x (\gamma(\mu + \Sigma x^\epsilon))^\top - \gamma(\mu\mu^\top + \Sigma) \\
&= \gamma(\mu + \Sigma x^\epsilon)(\mu + \Sigma x^\epsilon)^\top + \gamma\Sigma - \gamma(\mu\mu^\top + \Sigma) \\
&= \gamma\mu(\Sigma x^\epsilon)^\top + \gamma(\Sigma x^\epsilon)\mu^\top + \gamma(\Sigma x^\epsilon)(\Sigma x^\epsilon)^\top .
\end{aligned} \tag{S34}$$

C.4.3 Combining results to obtain update equations of the Synaptic Filter

With the results for the observation terms, i.e., Equations (S33) and (S34), the variance update Equation (S22) simplifies considerably. Indeed, the two last terms of Equation (S22) can be simplified as follows:

$$\begin{aligned}
&\frac{\mathrm{cov}(ww^\top, g) - \mu\mathrm{cov}(w^\top, g) - \mathrm{cov}(w, g)\mu^\top}{\gamma}\mathrm{d}\delta - \frac{\mathrm{cov}(w, g)\mathrm{cov}(w^\top, g)}{\gamma^2}\mathrm{d}N \\
&= (\Sigma x^\epsilon)(\Sigma x^\epsilon)^\top \mathrm{d}\delta - (\Sigma x^\epsilon)(\Sigma x^\epsilon)^\top \mathrm{d}N \\
&= -\gamma(\Sigma x^\epsilon)(\Sigma x^\epsilon)^\top \mathrm{d}t.
\end{aligned} \tag{S35}$$

We obtain the update equations for the Synaptic Filter and the Diagonal Synaptic Filter (for which Σ is a diagonal matrix), by substituting the results of prior and observations, i.e., Equations (S26) to (S28), (S33) and (S34), into the update Equations (S21) and (S22) for the moments. In addition, we use $y := \mathrm{d}N/\mathrm{d}t$ to switch to standard ODE-notation:

$$\dot{\mu} = \Sigma x^\epsilon (y - \gamma) + \tau_{\mathrm{ou}}^{-1}(\mu_{\mathrm{ou}} - \mu), \tag{S36}$$

$$\dot{\Sigma} = -\gamma(\Sigma x^\epsilon)(\Sigma x^\epsilon)^\top + 2\tau_{\mathrm{ou}}^{-1}(\Sigma_{\mathrm{ou}} - \Sigma), \tag{S37}$$

where $y := \mathrm{d}N/\mathrm{d}t$. In the Main Text, the determinism parameter β scales the input variable x^ϵ.

C.5 Block diagonal projection filtering

The filter obtained in Equations (S36) and (S37) can be seen as a projection filter on the subspace of multivariate normal distributions with full covariance matrices. However, computing the evolution of this full covariance matrix is both computationally expensive and not biologically plausible. As a consequence, we derive here simpler filters.

In particular, the purpose of this section is to calculate the projection of the vector field expressed in Equation (S37) on the linear space $\mathrm{Sym}\,(d)$ of $d \times d$ symmetric matrices onto the subspace $\mathrm{Block}\,(b_1, ..., b_m)$ of block-diagonal matrices, where m is the number of blocks, $b_1, ..., b_m \geq 1$, where $b_1 + ... + b_m = d$.

Before effectively deriving the block-diagonal projection filter, let us recall some properties of block diagonal matrices. Let $A_{\mathrm{block}}, A'_{\mathrm{block}} \in \mathrm{Block}\,(b_1, \ldots, b_m)$ be block diagonal matrices and $B_{\mathrm{offblock}} \in \mathrm{OffBlock}\,(b_1, \ldots, b_m)$ be an off-diagonal block matrix. Then we have that:

$$A_{\mathrm{block}} \cdot A'_{\mathrm{block}} \in \mathrm{Block}\,(b_1, \ldots, b_m) \tag{S38}$$

$$A_{\mathrm{block}} \cdot B_{\mathrm{offblock}} \in \mathrm{OffBlock}\,(b_1, \ldots, b_m) \tag{S39}$$

The metric to be used for the projection is the metric induced on $\mathrm{Sym}(d)$ by pulling back the Fisher-Rao metric[2] on the manifold of d-variate normal distributions along the map $\Sigma \mapsto \mathcal{N}(0, \Sigma)$. This metric is given by (see [10], Eq. 23):

$$\langle A, B\rangle_\Sigma = \frac{1}{2}\mathrm{tr}\left(\Sigma^{-1}A\Sigma^{-1}B\right). \tag{S40}$$

where $A, B \in \mathrm{Sym}(d)$. We observe that the tangent space $T_\Sigma\mathrm{Sym}(d)$ at $\Sigma \in \mathrm{Block}(b_1, ..., b_m)$ is isomorphic to $\mathrm{Sym}(d)$, i.e. $T_\Sigma\mathrm{Sym}(d)$ can be expressed as an orthogonal sum:

$$T_\Sigma\mathrm{Sym}(d) = \mathrm{Block}(b_1, ..., b_m) \oplus \mathrm{OffBlock}(b_1, ..., b_m), \tag{S41}$$

i.e. if we write $A, B \in \mathrm{Sym}(d)$ as $A = A_{\mathrm{block}} + A_{\mathrm{offblock}}$ and $B = B_{\mathrm{block}} + B_{\mathrm{offblock}}$, we have by the properties of the trace that:

$$\langle A, B\rangle_\Sigma = \langle A_{\mathrm{block}}, B_{\mathrm{block}}\rangle_\Sigma + \langle A_{\mathrm{offblock}}, B_{\mathrm{offblock}}\rangle_\Sigma. \tag{S42}$$

Indeed, to show Eq. (S42), it is sufficient to show that $\langle A_{\mathrm{block}}, B_{\mathrm{offblock}}\rangle_\Sigma = 0$. Since Σ is assumed to be block-diagonal, then Σ^{-1} is also block-diagonal. From Eqs. (S38) we have that $\Sigma^{-1}A_{\mathrm{block}}$ is block-diagonal and from Eq. (S39), we have that $\Sigma^{-1}B_{\mathrm{offblock}}$ is an off-diagonal block matrix. As a consequence $\Sigma^{-1}A_{\mathrm{block}}\Sigma^{-1}B_{\mathrm{offblock}}$ is an off-diagonal block matrix and therefore its trace is zero.

Let $\Sigma^{(i)}, i = 1, \ldots, k$, with $k = d(d+1)/2$ be an orthonormal basis of $\mathrm{Sym}(d)$, i.e. $\left\langle \Sigma^{(i)}\Sigma^{(j)}\right\rangle_\Sigma = \delta_{ij}$, such that $\mathrm{span}(\Sigma^{(1)}, \ldots, \Sigma^{(k_1)}) = \mathrm{Block}(b_1, \ldots, b_m)$ and $\mathrm{span}(\Sigma^{(k_1+1)}, \ldots, \Sigma^{k}) = \mathrm{OffBlock}(b_1, \ldots, b_m)$, where $k_1 = \sum_{i=1}^m b_i(b_i+1)/2$.

Because of the orthonormality of the basis set $\{\Sigma^{(i)}\}_{i=1}^k$, any matrix $A \in \mathrm{Sym}(d)$ can be written as:

$$A = \underbrace{\sum_{i=1}^{k_1}\left\langle \Sigma^{(i)}, A\right\rangle \Sigma^{(i)}}_{:=A_{\mathrm{block}}} + \underbrace{\sum_{j=k_1+1}^{k}\left\langle \Sigma^{(j)}, A\right\rangle \Sigma^{(j)}}_{:=A_{\mathrm{block}}} \tag{S43}$$

and the projection operator onto the subspace of block diagonal matrices can be expressed as:

$$\mathrm{Proj}_{\mathrm{Block}}(A) = \sum_{i=1}^{k_1}\left\langle \Sigma^{(i)}, A\right\rangle_\Sigma \Sigma^{(i)} \tag{S44}$$

for a matrix $A \in \mathrm{Sym}(d)$. As a consequence, the projection of the infinitesimal change in covariance matrix $\mathrm{d}\Sigma = \mathrm{d}\Sigma_{\mathrm{block}} + \mathrm{d}\Sigma_{\mathrm{offblock}}$ is given by:

$$\begin{aligned}\mathrm{Proj}_{\mathrm{Block}}(\mathrm{d}\Sigma) &= \mathrm{Proj}_{\mathrm{Block}}(\mathrm{d}\Sigma_{\mathrm{block}}) + \mathrm{Proj}_{\mathrm{Block}}(\mathrm{d}\Sigma_{\mathrm{offblock}}) \\ &= d\Sigma_{\mathrm{block}}\end{aligned} \tag{S45}$$

From this, it follows that the projection can be performed by simply cropping the off-block entries of $\dot\Sigma$. If we denote the blocks of $\Sigma \in \mathrm{Block}(b_1, ..., b_m)$ and Σ_{ou} (disregarding off-block entries) as Σ_i and $\Sigma_{i,\mathrm{ou}}$ and the corresponding subvectors of x as x_i, we obtain that the blocks of the projection of $\dot\Sigma$ simply evolve as

$$\dot\Sigma_i = -\gamma\Sigma_i x_i^\epsilon x_i^{\epsilon\top}\Sigma_i - \frac{2}{\tau_{\mathrm{ou}}}(\Sigma_i - \Sigma_{i,\mathrm{ou}}). \tag{S46}$$

Note that when $m = n$, we have m blocks of size 1×1 and we recover the Diagonal Synaptic Filter (see also Section C.2).

[2]Note that the Fisher-Rao metric is the only Riemanian metric (up to rescaling) that is invariant under sufficient statistics.

D Optimal learning rate for the gradient rule

In Fig 2 we compare the performance of the gradient rule with the one of different synaptic filters. In order to state that the synaptic filters outperform the gradient rule, we must select the optimal learning rate and therefore minimise the MSE as a function of the learning rate.

Our strategy was to evaluate the MSE at 10 log-spaced values for the learning rate in the interval $[2^{-9}, 2^{0}]$. The choice of the interval is motivated in the Main Text by noting that the learning rate replaces the variance in the Diagonal Synaptic Filter. Fig S3 shows that the interval contains indeed the optimal learning rate.

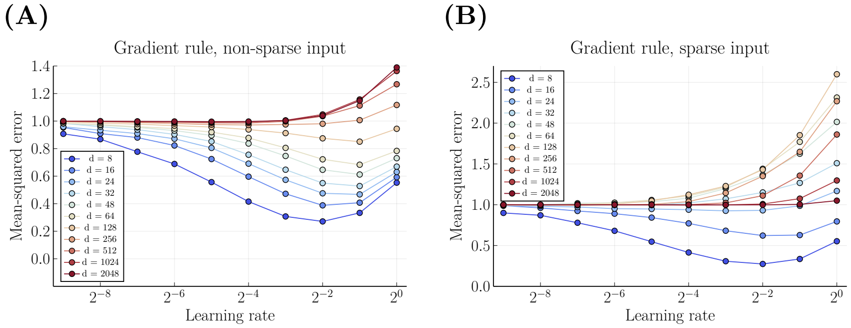

Fig S3. Optimisation of the learning rate for the gradient rule. **(A)** For each input dimension d (colored from blue to red), the MSE is computed as a function of the learning rate. The optimal learning is chosen as the one that minimises the MSE. The simulation results correspond to the non-sparse case, i.e. when all the input have the same firing rate. See Fig 2D. **(B)** Same as **(A)**, but for the sparse input case (see Fig 2E).

E Variables of the Synaptic Filter during simulated biological experiments

The Synaptic Filter can explain the biological phenomena of STDP and the negative correlation between homo- and heterosynaptic plasticity. The goal of this section is to show how the interaction of the variables of the Synaptic Filter produces the aforementioned biological observations. In the following, we show the time series of the mean μ_t and covariance matrix Σ_t, alongside the simulated protocols.

The STDP curve shown in the Main Text arises from a series of simulations with varying spike-timings. Fig S4A and 3B show Synaptic Filter variables for the cases $t_{\text{pre}} - t_{\text{post}} = \pm 10$ms, linked to the positive and negative STDP lobe respectively. The strength of potentiation in Fig S4A is related to the amount of presynaptic activation present when the postsynaptic spike occurs. The reason is that the product $y_t x_t^{\epsilon}$ is proportional to the update of the mean in Equation (S36). The strength of depression in Fig S4B depends on amplitude of the mean of the bias when the presynaptic spike occurs. The mean bias increases the expected firing rate γ_t, which modulates the update via the term $-\gamma_t x_t^{\epsilon}$. Thus the timescale of the negative lobe is directly associated with the prior timescale of the bias $\tau_{\text{ou,bias}}$, which controls how quickly the bias returns to its equilibrium value. From this mechanistic perspective on the STDP protocol, it becomes clear that the negative lobe depends on the presence of the bias. However, this result does not qualitatively depend on including the dynamics of the covariance matrix.

The simulations of the heterosynaptic plasticity protocol are similar to the STDP protocol; however, they include a preconditioning protocol and an additional synaptic weight, whose change in strength we label as heterosynaptic plasticity. Fig S5A and S4B

show Synaptic Filter variables for the cases $t_{\text{pre}} - t_{\text{post}} = \pm 10$ms. The preconditioning protocol consists of two presynaptic spikes at both inputs with minimal delay. This correlated input causes negative weight correlations via Equation (S37). When the pre-before-post, shown in Fig S4A, and post-before-pre, shown in Fig S4B, protocols are applied, the negative weight correlation between the first and the second weight leads to opposing directions of plasticity. Mathematically, this is manifested in the prefactor $\Sigma_t x_t^\epsilon$ in the updates, Equation (S36). When weight correlations are present, the covariance matrix converts presynaptic activation in the first weight into a non-zero coefficient in the second weight, i.e., the matrix prefactor mixes presynaptic activation between inputs. Thus, the results for heterosynaptic plasticity depend on the presence of weight correlations.

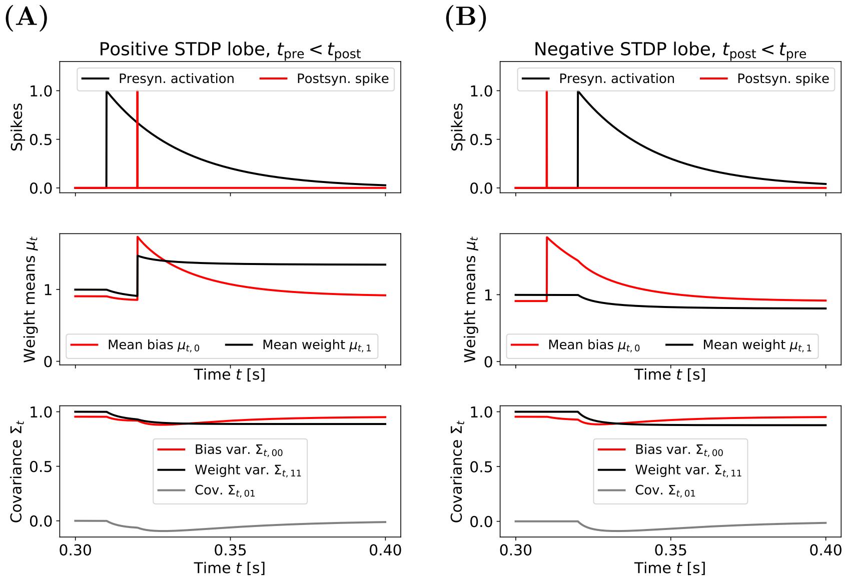

Fig S4. The dynamics of the variables of the Synaptic Filter during the STDP protocol. **(A, top)** shows a protocol for the positive lobe with a presynaptic spike (black) followed by a postsynaptic spike (red) with 10ms delay. The presynaptic activation paired with a postsynaptic spikes increases the mean value of the bias (red) and synaptic weight (black), shown in **(A, middle)**. The mean value of the bias returns to its equilibrium value on a timescale of $\tau_{\text{ou,bias}} = 25$ms. **(A, bottom)** shows that spiking activities reduces the elements of the covariance matrix. The variance of the bias returns quickly to its equilibrium value. **(B, top)** shows a protocol for the negative lobe. Importantly the depression of the mean weight (black) in **(B, middle)** is modulated by mean value of the bias (red). The smaller the delay of the presynaptic spike, the larger is the decrease in the mean weight.

F Negativity of weight correlations

In the Materials and Methods in the Main Text, we showed that the weight correlations, i.e., the off-diagonal elements of the covariance matrix Σ, are always negative in two dimensions. In the following, we show that for constant input $x_t^\epsilon \equiv x_0 = \text{const}$ the off-diagonal elements are negative for any d.

To show this, we represent the covariance matrix in the orthogonal, normalised basis: $\hat{x}_i^\top \hat{x}_j = \delta_{ij}$ for all $i, j \in (0, \ldots, d-1)$. The basis is chosen such that its first basis vector is parallel to the input vector: $\hat{x}_0^\top x_0 = ||x_0||^2$. In this basis, the covariance matrix is:

$$\Sigma = \sum_{i=0}^{d-1} a_i \hat{x}_i \hat{x}_i^\top \,, \tag{S47}$$

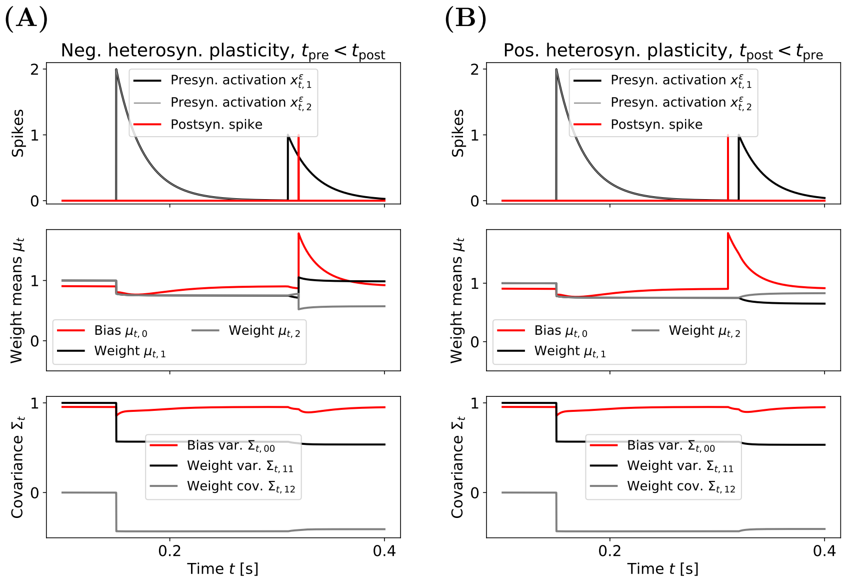

Fig S5. The dynamics of the variables of the Synaptic Filter during the heterosynaptic protocol with preconditioning. **(A, top)** shows the presynaptic activation (black, gray) of both synapses and the postsynaptic spikes (red) for a pre-before-post protocol. The initial waiting time of 100ms has been removed. **(A, middle)** shows the mean weights (black, gray) and the bias (red). The bias returns to its equilibrium value after the preconditioning protocol. At $t = 300$ms, the pre-post protocol is applied to the first weight, leading to potentiation and depression in the first and second mean weight respectively. **(A, bottom)** shows elements of the covariance matrix. The preconditioning protocol reduces the variance values. In particular, the covariance between the weights drops below zero. A non-zero value of the negative covariance causes the heterosynaptic plasticity. **(B)** shows a post-before-pre protocol. In contrast to the previous case, the arrival of a presynaptic spike at $t = 300$ms causes homosynaptic depression (black) and heterosynaptic potentiation (gray), as shown in **(B, middle)**.

where $a_i \geq 0$ because the covariance is positive semi-definite. Using this representation in the covariance update Equation (S37) and projecting the dynamics onto the basis yields update equations for the coefficients:

$$\dot{a}_i = \hat{x}_i^\top \dot{\Sigma} \hat{x}_i = -\delta_{i0} \gamma a_i^2 ||x_0||^2 - 2\tau_{\text{ou}}^{-1}(a_i - \sigma_{\text{ou}}^2), \tag{S48}$$

where we used $\Sigma_{\text{ou}} = \mathbb{1}\sigma_{\text{ou}}^2$ to obtain the second term. Equation (S48) has formal similarity with the variance updates of the Diagonal Synaptic Filter. However, because a_i are the coefficients of the decomposition in Equation (S47), the dynamics of the full covariance matrix are contained in them.

The initial condition of the covariance matrix is $\Sigma = \sigma_{\text{ou}}^2 \mathbb{1}$. Thus, the initial condition of the coefficients is $a_i = \sigma_{\text{ou}}^2$. It follows from Equation (S48) that the updates of the coefficients are non-positive $\dot{a}_i \leq 0$. The values of a_i decrease until a fixed point is reached. This implies, because the elements of the first basis vector are non-negative, $\hat{x}_{0,k} \geq 0$ for all $k \in (0, \ldots, d-1)$, that all elements of the covariance matrix, including the off-diagonals, decrease as well until the fixed points of the coefficients a_i are reached.

Empirically, we find that weight correlations are non-positive in the case of time-dependent inputs as well. The following analysis of a change from one static input to another supports this observation because it concludes that the first coefficient still has the fastest reduction rate after the change.

The time-dependent input vector is x_t^ϵ. Changes in the input vector correspond to a transformation of the basis vectors $\{\hat{x}_i\}$, used in Equation (S47). Under such transformations, the first basis vector $\hat{x}_t^\epsilon$ remains in the first quadrant because its entries are always non-negative. Without loss of generality, we assume that such a transformation is characterised by a rotation $\phi \in [0, \pi/2]$ in the plane of the first two

vectors of the basis and under the constraint that the first basis vector remains in the first quadrant. Thus, the first and second coefficient change as follows:

$$a'_0 = \cos(\phi)a_0 + \sin(\phi)a_1 \tag{S49}$$
$$a'_1 = -\sin(\phi)a_0 + \cos(\phi)a_1. \tag{S50}$$

From the analysis of the static case and the presence of an additional decay term in Equation (S48) for the first coefficient, we conclude that $a_0 < a_1$ before the change of the input vector occurs. Thus Equation (S49) implies that $a'_0 > a_0$, which leads to a faster decay of the first coefficient after the change of input. Equation (S50) implies that $a'_1 < a_1$, resulting in a slower recovery to the equilibrium value σ^2_{ou} than without the change in input. However, the effect of changing the basis vectors is neglected here.

Intuitively, the transformation, which corresponds to changes in the input, shifts the direction of maximal reduction of the covariance away from the direction in which the covariance has been maximally reduced under the previous input. Thus, the coefficient a_0 remains the one with the highest rate of reduction compared to the other coefficients.

Turning this argument into a proof in the case of time-varying inputs would require the investigation of all edge cases of the coefficient dynamics and is beyond the scope of this work.

References

1. Aitchison L, Latham PE. Synaptic sampling: A connection between PSP variability and uncertainty explains neurophysiological observations. arXiv preprint arXiv:150504544. 2015;.

2. Doucet A, Godsill S, Andrieu C. On sequential Monte Carlo sampling methods for Bayesian filtering. Statistics and computing. 2000;10(3):197–208.

3. Pfister JP, Dayan P, Lengyel M. Know thy neighbour: A normative theory of synaptic depression. In: Advances in neural information processing systems; 2009. p. 1464–1472.

4. Kushner HJ. On the differential equations satisfied by conditional probablitity densities of Markov processes, with applications. Journal of the Society for Industrial and Applied Mathematics, Series A: Control. 1964;2(1):106–119.

5. Kushner HJ. Dynamical equations for optimal nonlinear filtering. Journal of Differential Equations. 1967;3(2):179–190.

6. Brémaud P. Point processes and queues: martingale dynamics. vol. 50. Springer; 1981.

7. Minka TP. A Family of Algorithms for Approximate Bayesian Inference. PhD dissertation, Massachusetts Institute of Technology. 2001;.

8. Sugiyama M, Suzuki T, Kanamori T. Density-ratio matching under the Bregman divergence: a unified framework of density-ratio estimation. Annals of the Institute of Statistical Mathematics. 2012;64(5):1009–1044.

9. Kutschireiter A, Surace SC, Pfister JP. The Hitchhiker's guide to nonlinear filtering. Journal of Mathematical Psychology. 2020;94:102307.

10. Malagò L, Pistone G. Information Geometry of the Gaussian Distribution in View of Stochastic Optimization. In: Proceedings of the 2015 ACM Conference

on Foundations of Genetic Algorithms XIII. New York, NY, USA: ACM; 2015. p. 150–162. Available from:
https://dl.acm.org/doi/10.1145/2725494.2725510.
