# Supplementary figures and images for: Learning as filtering: Implications for spike-based plasticity
Source: PLoS Comput Biol. 2022 Feb 23;18(2):e1009721. doi: 10.1371/journal.pcbi.1009721 (PMC8865661; doi:10.1371/journal.pcbi.1009721)

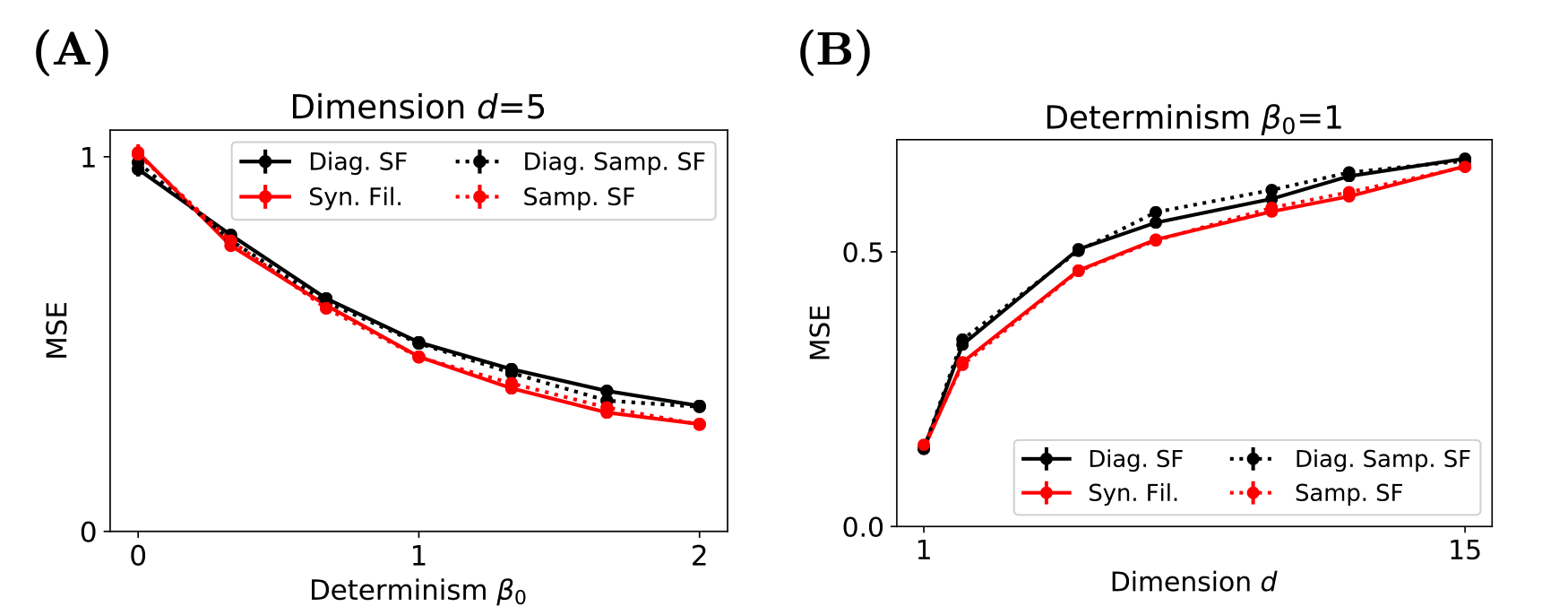

Supplement: S1 Text — S1 Fig. The Sampling Synaptic Filters have similar MSEs to their deterministic counterparts. S2 Fig. The first and second moments of the Synaptic Filter match the corresponding moment of the exact filtering distribution. S3 Fig. Optimisation of the learning rate for the gradient rule. S4 Fig. The dynamics of the variables of the Synaptic Filter during the STDP protocol. S5 Fig. The dynamics of the variables of the Synaptic Filter during the heterosynaptic protocol. (ZIP) [file pcbi.1009721.s001.zip › S1_Fig.tif]

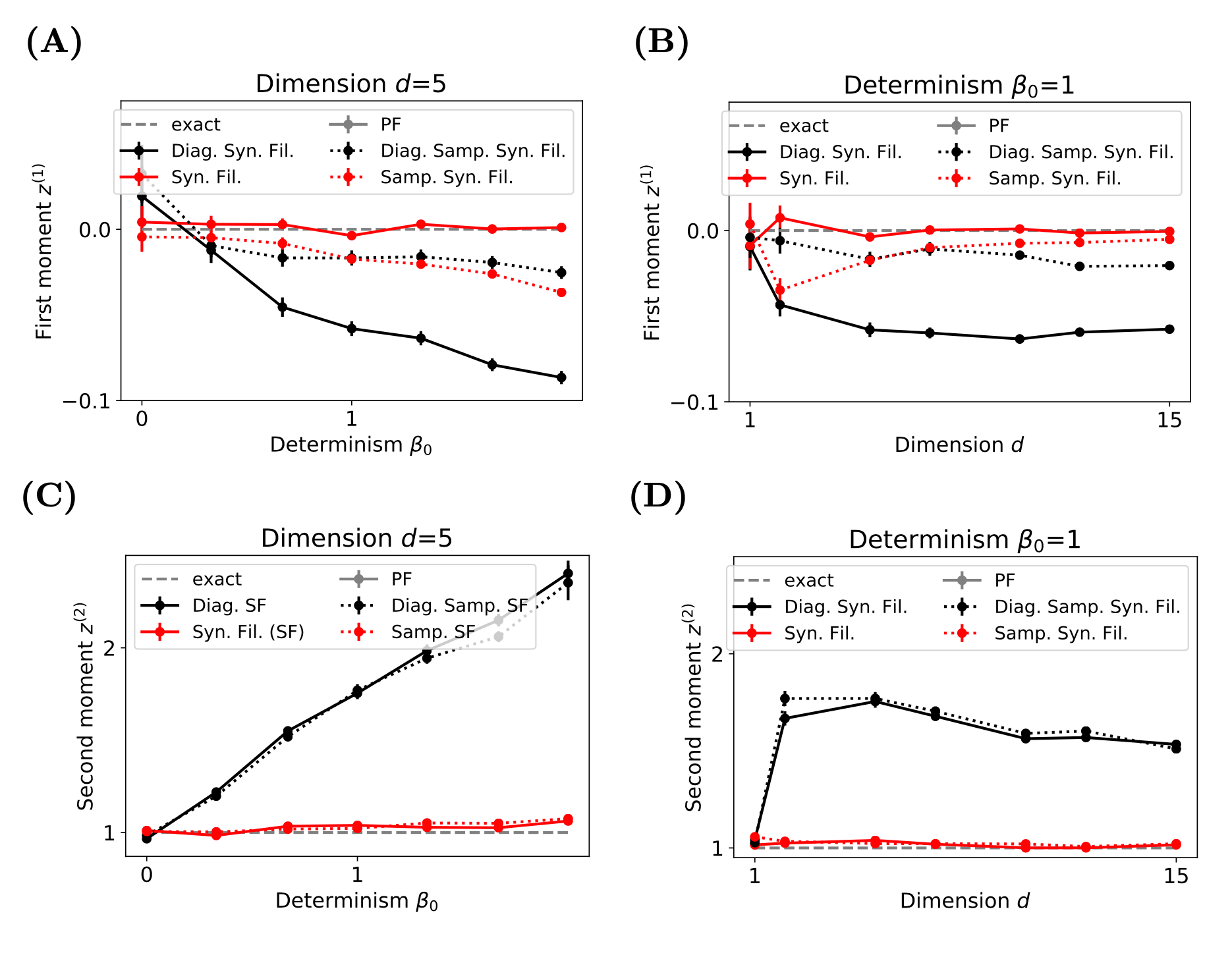

Supplement: S1 Text — S1 Fig. The Sampling Synaptic Filters have similar MSEs to their deterministic counterparts. S2 Fig. The first and second moments of the Synaptic Filter match the corresponding moment of the exact filtering distribution. S3 Fig. Optimisation of the learning rate for the gradient rule. S4 Fig. The dynamics of the variables of the Synaptic Filter during the STDP protocol. S5 Fig. The dynamics of the variables of the Synaptic Filter during the heterosynaptic protocol. (ZIP) [file pcbi.1009721.s001.zip › S2_Fig.tif]

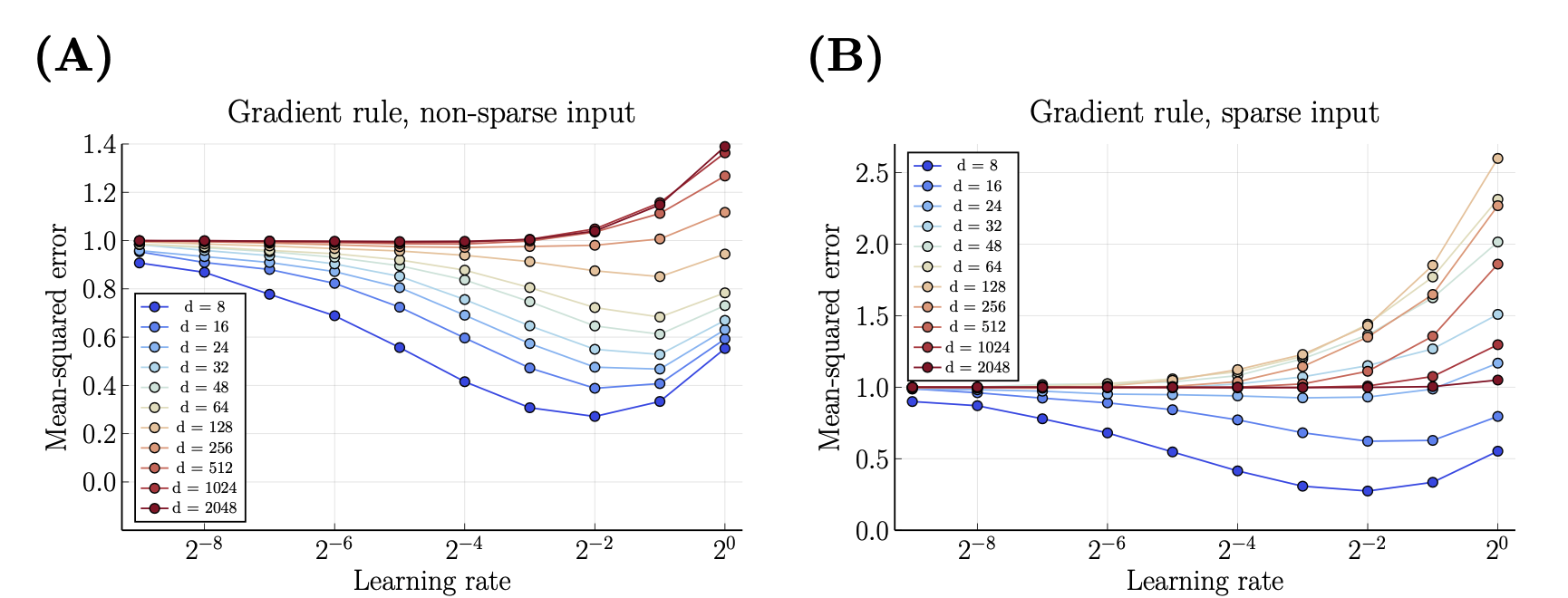

Supplement: S1 Text — S1 Fig. The Sampling Synaptic Filters have similar MSEs to their deterministic counterparts. S2 Fig. The first and second moments of the Synaptic Filter match the corresponding moment of the exact filtering distribution. S3 Fig. Optimisation of the learning rate for the gradient rule. S4 Fig. The dynamics of the variables of the Synaptic Filter during the STDP protocol. S5 Fig. The dynamics of the variables of the Synaptic Filter during the heterosynaptic protocol. (ZIP) [file pcbi.1009721.s001.zip › S3_Fig.tif]

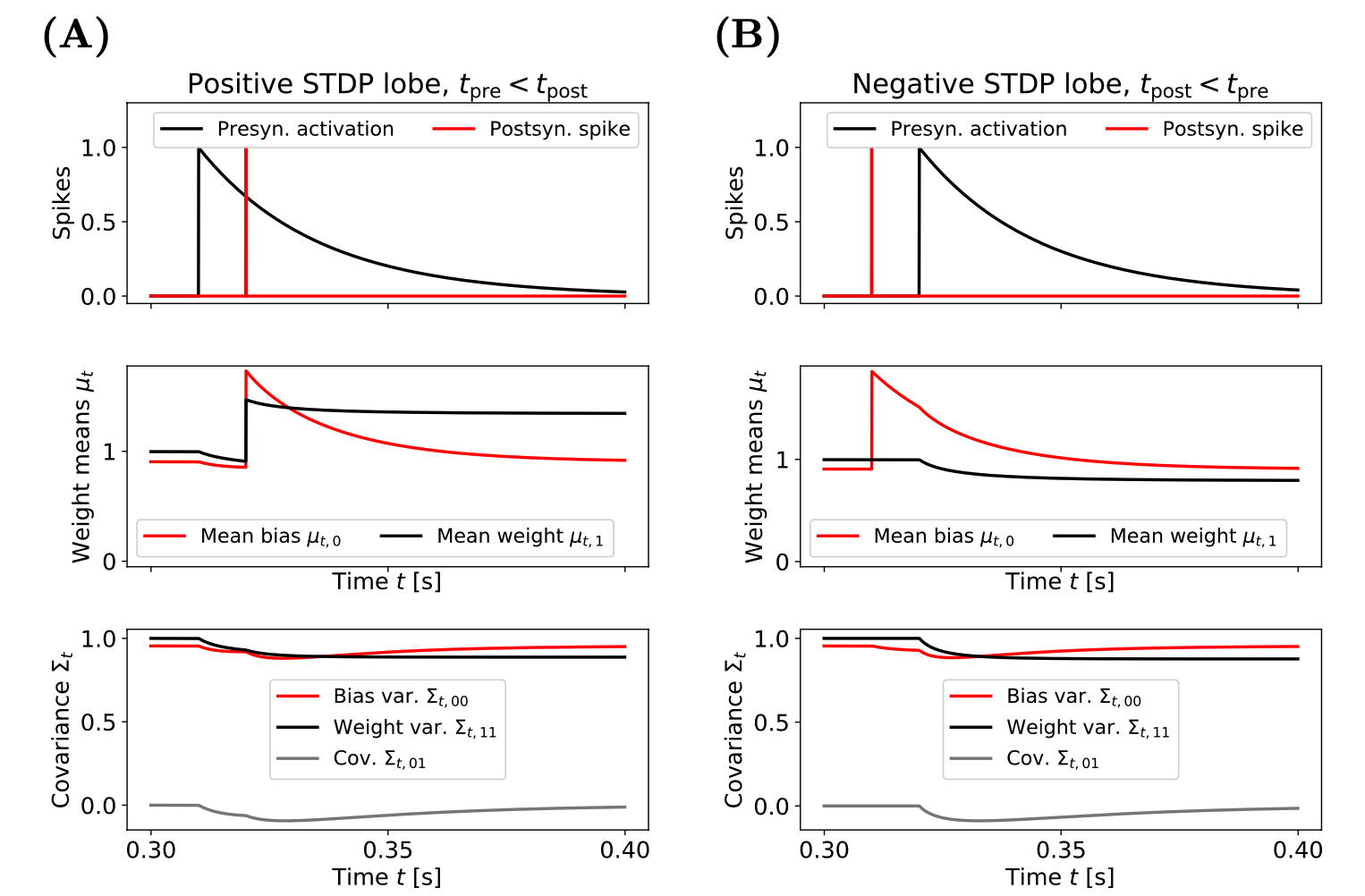

Supplement: S1 Text — S1 Fig. The Sampling Synaptic Filters have similar MSEs to their deterministic counterparts. S2 Fig. The first and second moments of the Synaptic Filter match the corresponding moment of the exact filtering distribution. S3 Fig. Optimisation of the learning rate for the gradient rule. S4 Fig. The dynamics of the variables of the Synaptic Filter during the STDP protocol. S5 Fig. The dynamics of the variables of the Synaptic Filter during the heterosynaptic protocol. (ZIP) [file pcbi.1009721.s001.zip › S4_Fig.tif]

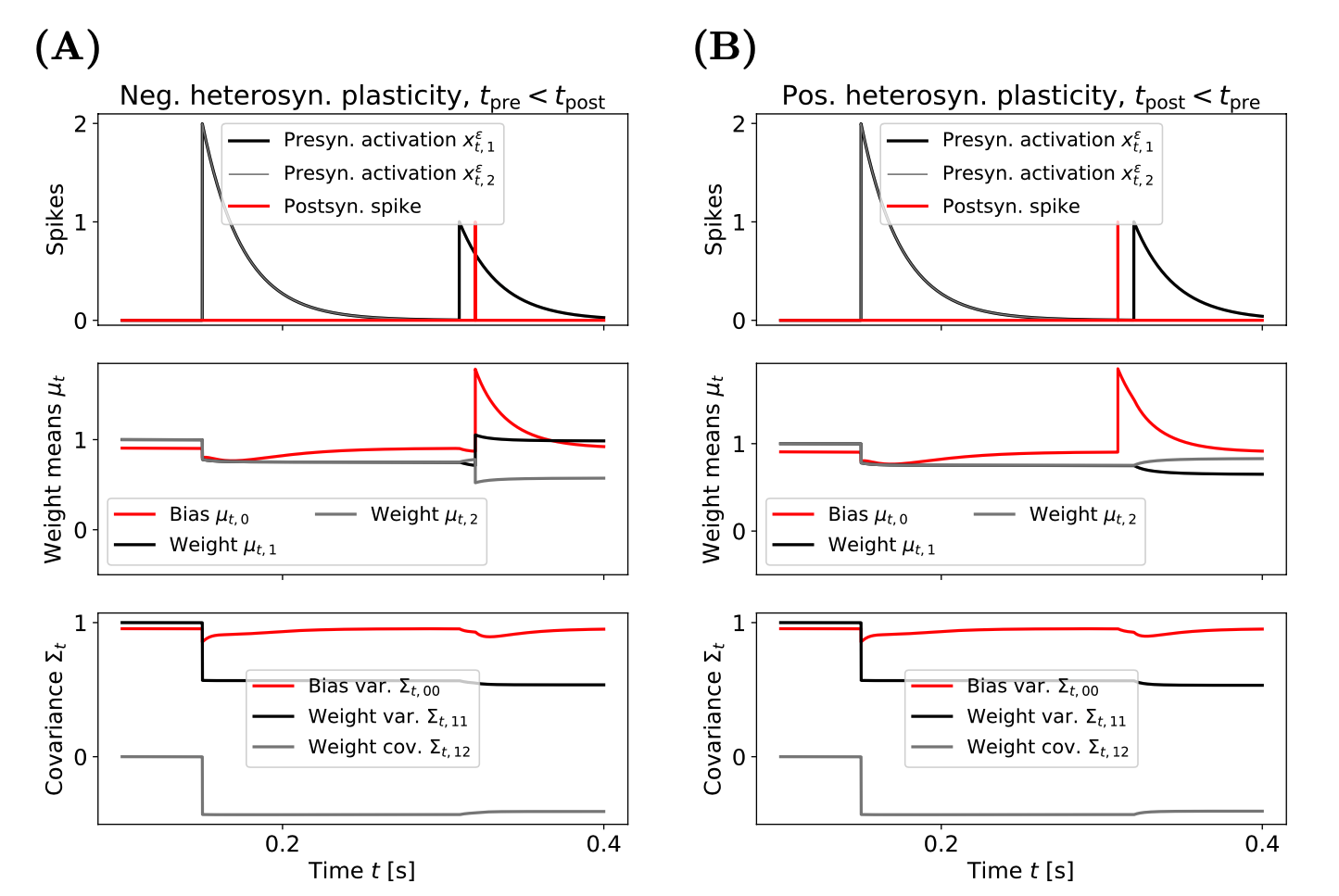

Supplement: S1 Text — S1 Fig. The Sampling Synaptic Filters have similar MSEs to their deterministic counterparts. S2 Fig. The first and second moments of the Synaptic Filter match the corresponding moment of the exact filtering distribution. S3 Fig. Optimisation of the learning rate for the gradient rule. S4 Fig. The dynamics of the variables of the Synaptic Filter during the STDP protocol. S5 Fig. The dynamics of the variables of the Synaptic Filter during the heterosynaptic protocol. (ZIP) [file pcbi.1009721.s001.zip › S5_Fig.tif]
