# Supplementary material for: Learning as filtering: Implications for spike-based plasticity
Source: PLoS Comput Biol. 2022 Feb 23;18(2):e1009721. doi: 10.1371/journal.pcbi.1009721 (PMC8865661; doi:10.1371/journal.pcbi.1009721)
Supplement: Attachment — Submitted filename: response_to_reviewers_v2.pdf [file pcbi.1009721.s002.pdf]

Response to Reviewers for the manuscript "Learning as filtering: implications for spike-based plasticity"

Jannes Jegminat and Jean-Pascal Pfister

Version: July 16, 2021

1 Reviewer 1

1.1 Summary

This papers interprets synaptic plasticity as a filtering process, tracking a time-varying generator. The system tries to infer the synaptic weights of the generator and find that under some condition the learning rule resembles STDP. This is a though-provoking study that would suit well in PlosCB.

It is also a technically competently carried out study. That said, I have my doubts if the results are really offering new insights to STDP. The authors correctly state that the depression lobe of STDP has not been well explained. But a simple explanation could be that biophysically it is an efficient way. Similarly, heterosynaptic competition is present in many models.

We thank the reviewer for having appreciated our paper both in terms of the change in paradigm it offers as well as its technical derivations.

Regarding the contribution to STDP and heterosynaptic plasticity, we want to stress the fact that in neuroscience, there are several levels of explanations that complement each other and therefore do not necessarily exclude each other. In this manuscript, we propose a normative model for synaptic plasticity which aims to understand synaptic plasticity from normative principle (i.e. learning as filtering). This should not and indeed does not prevent alternate explanations of STDP and heterosynaptic plasticity from biophysical considerations. Indeed biophysical models aim to explain phenomena (such as synaptic plasticity) as a function of the dynamics of its constituents (i.e. ion channels and vesicle releases.)

1.2 Major

(1) The paper is reasonably clear, but the split of information into Results, Materials and Methods, and Supplementary Information needs attention. As an indication of the poor organization is the fact the τ_m was set to 25 ms was mentioned three times.

To address this concern, we moved relevant information from the Materials and Methods to the Results; and we condensed the Materials and Methods section.

- To concentrate the information more, we moved the definition of the generative model from the Materials and Methods to the Section 2.1. In the new version, Equation (1-4) formally introduce the prior over the weights, the observation process and the membrane potential.
- When the generative model is introduced (Section 2.1), we no longer explain the scaling of β. The reason is that the scaling is only relevant for simulations across a range of dimensions. Therefore, we moved the explanation into the Section 2.2, in which the computational results are discussed.

- To contextualise the bias better, we mention it twice. First, after the definition of the membrane potential. Secondly, we provide a more technical definition when we explain our rationale for the STDP-experiments (Section 2.3).
- To clarify the meaning of all variables in the update Equations (5) and (6), we define the expected firing rate in Equation (7). Previously, it was placed in the Materials and Methods.
- The simulation details for the MSE results in Section 2.2 were previously scattered across three subsections in the Materials and Methods. They were concentrated into a single section (Section 4.3).

(2) In the setup the generative model is another single neuron with dynamic synaptic weights. In this case it is possible to derive the optimal update rule. (As an aside, the role of the zero mean and diagonal covariance in the generator should be discussed/explored.)

We agree with the reviewer that the specific choice of the generative model parameters could be discussed. Indeed, this is the case for any generative model and not only for the one considered here. Our reasoning is the following:

- the zero mean in the OU process give rise to a weight decay term in the learning rule that pushes weights towards zero. Such a weight decay towards zero is very common in the literature. It is computationally beneficial (Krogh and Hertz 1992) and has been also observed experimentally (Frey and Morris 1997).
- the prior covariance matrix Σ_{OU} has been set to be diagonal out of simplicity. Indeed assuming a specific covariance matrix Σ_{OU} with non-diagonal entries is technically possible but would require to justify that some inputs are correlated - a statement which is hard to make before any input is seen. Note also that instead of studying different prior covariance matrices, it is possible to study different preconditioning protocols (as it was done in Fig. 4) which precisely induce different correlation structures in Σ.

We clarified this in the main text by adding the following sentences in Section 2.1 (140ff): "The choice of parameters can also be motivated from the perspective of the resulting learning rule. Zero mean yields a weight decay on the time scale τ_{ou}. Weight decay has been observed experimentally (Frey and Morris, 1997). A diagonal covariance matrix reflects the assumption that weights are not correlated in the absence of inputs. As we show in Fig. 4 one way to study the effect of non-diagonal covariances on learning is by preconditioning with a spiking protocol of highly correlated inputs."

(3) It was not clear to me how this generalizes where the neuron tries to fit a non-linear model with dynamic parameters, where is perhaps not possible to derive the optimal learning rules. There must be benchmarks for such systems, I would imagine.

It is correct that the number of cases that are analytically tractable is very small. For example, the exponential gain function associated to an OU process - as considered in our manuscript - is tractable. Another tractable solution is sigmoidal transfer function. The nice aspect of the exponential gain function is that it has some experimental support (Jolivet, Rauch, Lüscher, and Gerstner 2006).

For arbitrary nonlinear gain functions, it is possible to perform a Taylor expansion of the log posterior density up to second order and obtain some approximate filter (see Eqs 2.7-2.10 in (Eden, Frank, Barbieri, Solo, and Brown 2004)). We added a footnote in the main text after Eq. 3.

(4) It also raises the question how the parameters of the learning rule (1) and (2) should be learned when they are not known.

We agree with the reviewer that the question of parameter learning deserves attention. There are however a few remarks we want to make.

First, any learning rule contains parameters. Those parameters are usually set and not optimised. For example in a classical Hebbian learning rule, the learning rate is typically set to some value and rarely optimised.

Secondly, the model parameters are technically learnable through maximum likelihood training (see (Surace and Pfister 2019) for some convergence results). However any parameter learning will also depend on other parameters (e.g. learning rate for ML or metaparameters for parameter inference).

So at the end of the day, we have to live with some parameters that we will not optimise.

(5) The time-scale of the STDP depression lobe seems to be related to the time-scale of the bias ($\tau_{ou,bias}$) (which would have been nice to be shown in a plot). The whole argument matching STDP to filtering hangs therefor on the rather adhoc assumptions that this is somewhere between 10 and 100ms. This needs more motivation.

In Section 2.3, we added a Fig 3C. The new figure shows τ_{LTD} as a function of τ_{bias} where τ_{LTD} is defined such that the learning window $W(-\tau_{\mathrm{LTD}}) = b + a/e$ where b is the baseline and a is the maximal amplitude of LTD.

We agree with the reviewer that there is some freedom on how to choose $\tau_{\mathrm{ou,bias}}$. It should be noted however that there is a rationale for constraining $\tau_{\mathrm{ou,bias}}$ to lie between 10 and 100ms. Indeed, if the bias represents the contribution from a set of randomly firing inputs:

$$b(t) = \sum_{j=1}^{m} w_j x_j^{\epsilon}(t) \tag{1}$$

where $x_j(t)$ is the spike train of input neuron j with Poisson firing rate $\rho_j(t)$ and $x_j^{\epsilon}(t)$ is the convoluted spike train (where the convolution kernel $\epsilon(s)$ has a time constant $\tau_\epsilon = 25$ ms). In the limit where m is large, b can be approximated as diffusion process $\tilde{b}$. If $\rho_j = \rho_0$, then the diffusion process $\tilde{b}$ is actually an Ornstein-Uhlenbeck process with time constant $\tau_{\tilde{b}} = \tau_\epsilon$. When the input rates are also fluctuating with a time constant τ_ρ, then the autocorrelation function will be characterised by two time constants: τ_ϵ for the fast decays and τ_ρ for the slower decays - which could typically be on the order of 100 ms.

So in summary, it is indeed reasonable to assume that $\tau_{\mathrm{ou,bias}}$ is somewhere between 10ms and 100ms. We added a shorter version of this argument in Section 2.3, L289.

(6) Does the STDP curve depend on firing rate (as has been observed)?
We appreciate this relevant comment. Before using a model to make specific predictions, we have to evaluate whether those predictions are robust or not. For example, robust predictions from our model are that pre-post spike pairs should lead to potentiation and that the extent of the LTP lobe should match the time scale of the EPSP. These predictions are robust because they don't depend on the specific choice of the gain function g nor do they critically depend on the specific choice of the model parameters.

Other predictions may be more fragile. Indeed, during higher frequency stimulations, the outcome of the competition between LTP (that comes from pre-post pairs) and LTD (that arise from post-pre pairs) will strongly depend on the choice of parameters. It is very likely that there exists some parameter choice for which the frequency dependence would resemble the one found experimentally. Indeed, (Brea, Senn, and Pfister 2013) (see their Fig. 5B) found such a frequency dependence for the gradient-based model where a very specific gain function (i.e. a rectified quadratic gain function) had to be assumed. Since all our results are derived from an exponential gain function and since we wanted to focus on robust predictions, we didn't include a frequency-dependence prediction and comparison with experimental data.

1.3 Minor

- Particle filtering is mentioned without introduction.

→ We removed all the references to the particle filter in the main text and described it in more details in the SI (section B.3). The particle filter is now only used as a reference filter for the moments computation in Fig. S2.

- MAP is undefined.

→ The paragraph including MAP has been actually removed

- In 2.2 it says that $\beta = \beta_0/d$, but in 2.1 $\beta = \beta_0/\sqrt(d)$

→ The typo was corrected.

- There is an edited volume of proceedings by Haykin from the late 90s(?) connecting filtering to neural networks.

→ We added a reference to the book of S. Haykin in the introduction.

2 Rev 2

2.1 Summary

Many normative models of learning use optimization of a cost function as the core principle. This framework typically ignores parameter uncertainty and the varying nature of the statistical environment. In this manuscript, the authors propose to employ the framework of nonlinear Bayesian filtering as an alternative. They call their approach the Synaptic Filter. In a nutshell, this approach infers the parameters of a Gaussian Assumed Density Filter (= Synaptic Filter), which is an approximation to the exact filtering distribution that gives the probability of the weights given the data. In so doing, they get updating equations for the mean and the covariance of the Gaussian filter; updates of the mean play the role of a standard learning rule, whereas the covariance updates do not typically appear in models.

Using this framework with a Poisson observation model and a Ornstein-Uhlenbeck state model, the authors reproduce well-known properties of synaptic plasticity (STDP rule and the heterosynaptic plasticity with preconditioning) as well as predicting a novel spike-timing dependent effect, this time on the variance of the synaptic weight. Interestingly, their model also suggests an origin for the LTD lobe in STDP. Overall, the authors claim that the Synaptic Filter both benefits predictive performance and brings out interesting conceptual points.

Assumed Density Filters have been studied in the context of Bayesian neural networks for many years as powerful alternatives to standard gradient descent (e.g., Ghosh... Yedidia, AAAI, 2016 for a recent example). In (computational) neuroscience, similar approaches have been used, for instance in the context of the synaptic sampling hypothesis (Aitchison & Latham, arXiv, 2015). The main contributions of the present manuscript are (1) to study synaptic plasticity in the general context of nonlinear Bayesian filtering and, mostly, (2) to apply this framework to Poisson neurons in order to unveil interesting aspects of synaptic plasticity that have been overlooked when studied in other contexts (namely, from an optimization point of view).

My general appreciation of this manuscript is positive, although I do have some points I would like the authors to address and clarify. The manuscript does belong to Plos Comp Biol for its novel interpretation of the LTD lobe, its description of heterosynaptic plasticity and its discussion of the STDP of variance, as well as for (implicitly) advocating for a more general use of nonlinear filtering when studying synaptic models.

Note that I'm not an expert of nonlinear filtering models and that I'm more used to dynamical and optimization-based models of plasticity.

2.2 Major points

(1) Variability of the environment The authors suggest that one of the drawbacks of (pointwise) optimization-based learning is that the variability of the environment is poorly accounted for.

However, I wonder how actually variable is the environment represented by the ground truth weight model (the OU process). Indeed, the ground truth model is a OU process with constant mean and variance, hence stationary. Wouldn't a time-dependent mean and variance be more appropriate to instantiate a changing environment? Along that same vein, perhaps plotting the weight dynamics of the gradient-based model in Fig. 1D would help clarify the expected dynamical differences between Bayesian inference and "pointwise" optimization.

There are actually many levels of variability. The least variable model (that we could call the 0th variability level) assumes that the ground truth weights are constant ($w = \text{cst}$). This assumption is at the core of the vast majority studies on learning.

The first level of variability assumes that the ground truth weights follow some diffusion process where the parameters are assumed to be constant. This is the case considered in the present manuscript. The ground truth weights are assumed to follow an OU process where the parameters μ_{ou} and Σ_{ou} are assumed to be constant.

We agree with the reviewer that in principle we could also consider a higher level of variability (i.e. 2nd variability level) which would assume that the parameters μ_{ou} and Σ_{ou} are themselves variable.

However, the immediate next question would be: why stopping at the 2nd level and why not consider up to the nth level? Since we believe that science must progress step by step, we think that it is reasonable to limit the present study to the first order level and then leave the 2nd level variability with its bag of interesting associated questions (e.g. how to find a biological plausible learning rule for such a hierarchical generative model?) to subsequent studies.

As requested by the reviewer, we added the gradient rule on Fig 1D. At the end of Section 2.1, L155: "A gradient rule (gray) with well chosen but fixed learning rate is shown for comparison. Unlike the Synaptic Filter, it cannot adjust its updates based on the remaining uncertainty around the hidden weight."

(2) Unfair comparison with gradient descent The authors argue that the Synaptic Filter might have a better predictive power than gradient descent (GD). To support this, they studied the predictions and performance under different models. My understand is that SF is more efficient than GD in most cases. However, SF seemingly has indirect "insider information" about the ground truth model, given the selected OU process and the Gaussian Assumed density filter. One would expect that a larger statistical mismatch (a larger KL divergence between the exact filtering distribution and the approximation) would be more problematic for SF, even with matching dimensions of the inputs. Also, when there is a model mismatch in term of the input dimensionality, Fig. 2E suggests that GD will eventually outperform SF (this is not shown in Fig. 2E and thus the comment at line 294 is unsubstantiated). The authors do argue (around line 224) that with sparse inputs the effective input dimension might be smaller than the expected 10000 synapses per neurons in the brain, but their operating of 1 spike/membrane time constant does not account for that. I would suggest to i) explore a higher dimension in Fig. 2E to show the point where GD outperform SF and ii) perhaps testing one sparse-input (a few Hz) model at high dimension (optional, but could be interesting). Showing the prediction for GD in Fig. 1D (as in the above point) could also help clarify the differences between the algorithms.

Regarding the comparison between the SF and the GD, let us make 2 remarks:

We agree that the SF has "insider information" in the sense that it has access to parameters of the generative model ($\mu_{\text{ou}}, \Sigma_{\text{ou}}, \tau_{\text{ou}}$)). However, it shouldn't be forgotten that for the comparison, one important parameter of the the GD learning rule, namely the learning rate, is optimised to give the best performance. So we could also argue that this optimisation gives a competitive advantage to the GD learning rule.

Note also that in the limit $\beta \to 0$, the exact filter is actually a Gaussian filter and therefore the Assumed Density filter is exact. The mismatch between the approximate filter and the exact filter increases with β, so it might be that when β is really large, the gradient rule outperforms the SF.

We decided to follow the reviewer's suggestion by pushing simulations to higher dimensions as well as studying the sparse input limit. We rewrote all the code in Julia to speed up simulations

and gained several orders of magnitude in simulation speed. In this way, we managed to compute the MSE for higher dimensions (see new Fig. 2D and 2E). We found that:

- The synaptic filter is relevant (i.e. $MSE < 1$) in high dimensions for a certain regime of parameters (see new Fig. 2D and E). In particular, the time constant of the Ornstein-Uhlenbeck process needs to be large.
- All three filters (dSF, bSF and full SF) are nicely consistent with each other when the input firing is low (sparse input regime). When the input firing rate is large, the performance between the different filters become more apparent (see new Fig2C).

We also decided to remove the old Figures 2D and 2E. See our response to Reviewer 3 section 3.2 paragraph 6 for our rationale for this choice.

(3) More equations in main text I do understand the desire to remove "clutter" from the main text by putting equations in the methods. However, my understanding of the paper did suffer from the lack of precise definitions of some quantities. First and foremost, the bias. Indeed, $\omega_{t,0}$ appears for the first time at line 203 but it was not defined before. Secondly, the expected firing rate γ_t should also appear somewhere after Eq. 2. So, I would suggest to put in the main text the equation for u (emphasizing the bias) and the expression for γ_t.

- We agree that the key mathematical definitions and results should be more concentrated in the main text. As part of the reorganisation of the manuscript, we moved the definitions of the membrane potential and posterior firing rate into the main text.
- To remove clutter, we moved the discussion of how to scale β with dimensions to computational results Section 2.2.
- The option to include a bias is now mentioned after the definition of the membrane potential, Equation (4). Because we did not include a bias in the new performance simulations (Section 2.2), we reintroduce the bias slightly more formally in Section 2.3, when it is actually used (L280).

2.3 Minor points

- The authors should refer to the supplementary figures in the main text where appropriate (e.g., refer to Fig. S4 when discussing STDP). Also, in the materials and methods section, they talk about particle filtering without properly referring to the supplements.

→ We removed all the references to the particle filter in the main text and now describe it in more details in the SI (section B.3). The particle filter is now only used as a reference filter for the moments computation in Fig. S2. We also added explicit references to all the supplementary figures.

- The authors use a comparatively fast time scale for the bias in the paired plasticity protocol. Since they associate this bias to excitability, which encompasses several aspects of neuron spiking, could they discuss what biophysical mechanisms they have in mind (after-hyperpolarization?) for it?

→ We assume indeed a fast time scale for the bias. Regarding the rationale behind this fast time scale, see our response to Reviewer 1, section 1.2, 5th paragraph.

We would like to stress that the assumption of a bias in the generative model does not mean the bias has to be implemented biophysically. Actually, only the posterior mean and variance of the bias appear in the update equations, not the bias itself. In the scope of our model, the mean and variance of the bias cannot influence the output spiking. This is why they cannot be linked to the neuron's excitability. In the current version, all mentions of neuronal excitability related to the bias were removed.

- Do the authors think this framework could reproduce other known aspects of synaptic plasticity, like the frequency dependence?

→ This is actually a very good point that was also proposed by reviewer 1. Please see our response to reviewer 1 in the last paragraph of section 1.2

- Beta in the exponential gain function is constantly referred to as "determinism" parameter. Since the model is purely stochastic, this name is quite odd. Please consider calling it "slope" parameter (or something of sort) instead.

→ The parameter β scales the membrane potential and therefore affects the firing rate of the Poisson neuron. In the limit of large β, the neuron fires deterministically in the sense that if the membrane potential is below 0, the neuron does not fire whereas if the membrane potential is above 0 it fires with certainty. We find that the alternative name, "slope", is not suitable for an exponential. Since previous studies called it also "determinism" (e.g. (Pfister, Dayan, and Lengyel 2010)), we think the name "determinism" parameter is appropriate. To explain our naming convention, we added the following sentence after Eq 3: "The determinism parameter β controls how strongly changes in the membrane affect the firing rate. In the limit $\beta \to \infty$, the neurons firing rate is deterministic, i.e., if the membrane potential is below 0, the neuron does not fire whereas if the membrane potential is above 0 it fires with probability 1."

- Lines 236-237: "a low MSE does not necessitate poor predictions." What did you mean here (esp. the word necessitate sounds out of place)? Moreover, a high MSE performance implies a low MSE. Please consider clarifying the many instances of mentioning MSE to make sure that no confusion remains.

→ We clarified several mentions of "MSE performance", e.g., in the first paragraph of the SI: "compatible with MSE performance" to "compatible with good MSE performance (low MSE values)". The sentence mentioned by the reviewer has been removed from the manuscript.

- Lines 240-242: "In case of [. . .] in detail by the brain." I don't think this is the issue here. I would say it is rather that no ground truth for the weights is available, only spiking activity supposed to come from that putative ground truth, whose dimensionality could eventually be known (connectomics) but not in every single neurons.

→ We thanks for the reviewer for the nice suggestion. Because the manuscript has been reorganised around the question of how the Synaptic Filter could work in high dimensions, we dropped the section on predictive performance, part of which the reviewer was referring to.

- I think the authors should "shamelessly" push their own tutorial on nonlinear filtering, especially in the introduction.

→ We appreciate this comment and now cite our tutorial in the introduction.

- I did not follow the discussion of section 5.6 on the negativity of the weight correlation. Please make sure the equations are correct; it felt like you were considering variances and not the off-diagonal terms of the matrix.

→ Besides double checking the equations, we added two sentences after Equation (S43) to clarify how a_i differ from the analysis of variances: "Equation (S43) has formal similarity with the variance updates of the Diagonal Synaptic Filter. However, because a_i are the coefficients of the decomposition in (S42), the dynamics of the full covariance matrix are contained in them."

- On line 178, typo in word "Diagnalised".

→ Corrected.

- Line 228: "Thus it is possible that learning in many brain systems takes place inside the learning regime." Sounds like a tautology. . .

→ The idea behind this unhappy formulation was that learning (low MSE) is not possible for all hyperparameter configurations (e.g. $\tau_{\text{ou}} \to 0$). The hyperparameter configurations that allow for learning was called "learning regime". The problematic sentence has been removed. Moreover, the new MSE results in Fig 2 (B-D) show clearly how MSE performance depends on relevant hyperparameters.

- Line 323: "Our rationale was that postsynaptic spiking predominately affects the bias wt,0, which represents the neuronal excitability. " Difficult to comprehend on first reading. More details would be welcome.

→ The reviewer's command made us realise that our mention of neuronal excitability was premature (see our answer above with respect to the question of how the bias could be implemented biophysically). We reframed the motivation for the STDP simulations (L278ff) around the question how the negative lobe arises. Additionally, we give details in a technical footnote: "Technically, the bias $w_{t,0}$ is a weight with constant, unit input, i.e., $x^{\epsilon}_{t,0} \equiv 1$. We use the first index $i = 0$ to represent the bias."

- I didn't quite follow the discussion in the paragraph starting at line 511. How is the fact that all recurrent neurons be visible change anything to the conditional independence of their spiking?

→ As the reviewer points out, our description wasn't very clear. We reformulated the whole paragraph as follows:
"The generalisation of the single neuron analysis to the case of a recurrent neuronal network is straightforward as long as all neurons in the recurrent population are visible. Indeed, when all neurons are visible, then the likelihood nicely factorises along the temporal dimension because of the chain rule and along the spatial dimension because of the conditional independence between the neurons (i.e the current spiking of neuron i at time t is only affected by the spiking history of all neurons up - but not including - time t). In the presence of hidden neurons, this factorisation is not possible anymore. Thus, the Synaptic Filter can be used to gain insights into learning dynamic weight distributions not only in a single neuron but also in the more complex setting of a recurrent neuronal network model, given that all neurons are visible."

3 Reviewer 3

3.1 Summary

I thank the authors for their submission. As my knowledge of the synaptic plasticity literature is rudimentary, my contribution as a reviewer will be to assess the technical accuracy of the manuscript and to evaluate it on its own terms rather than in reference to the large body of existing literature on synaptic plasticity.

The authors present the Synaptic Filter as an alternative to the optimization-based approaches for setting synaptic weights in learning tasks. The advantage of the Synaptic Filter is that it provides a principled way to track not just the mean value of the weights, but, unlike typical optimization based approaches, also the uncertainty about those weights. This not only improves performance in settings like regression by allowing averaging over weight uncertainty, it also easily accommodates settings in which environmental parameters change over time, which is of obvious relevance to real neural systems. Despite these clear benefits, the SF is only neuroscientifically relevant if the authors can make a convincing case that it is (a) neurally plausible and could indeed, at least in some reduced form, be implemented in the brain; and that (b) it provides significant advantages over other, perhaps less principled, approaches. Thus far I do not believe the authors have done so.

The authors make three specific claims about the advantage of the SF: That it provides a principled explanation of the LTD lobe of STDP; That it provides a principled explanation of heterosynaptic plasticity; and that it provides a testable prediction for the variance in weight changes in STDP experiments. I will address each of these points in turn, before discussing neural plausibility.

3.2 Major

(1) First, the positive: The SF predicts that in STDP settings, the variance of synaptic weight changes should drop as tpost approaches tpre from either direction, along with a principled explanation of why this should be so. I found this interesting, testable prediction to be a highlight of the paper. I merely wondered why, given that the authors had compared their predictions for heterosynaptic plasticity to experimental data, why they had not also done so here to test their prediction? This, while not absolutely necessary in my opinion, would significantly strengthen their paper.

We thank the reviewer for this relevant point. Indeed, we spent a decent amount of time analysing the STDP data from Sjostrom. Unfortunately, because of the level of the recording noise, we were not able to validate nor invalidate our predictions.

(2) The authors also show that in a simple STDP protocol, the SF can reproduce heterosynaptic plasticity similar to that observed in experiment. The principled nature of the SF really shines here in that it not only reproduces the effect, but also provides an explanation for heterosynaptic plasticity through the negative covariance induced between different synapses by the preconditioning protocol.

We thank the reviewer for this positive appreciation.

(3) The authors also claim that the Synaptic Filter reproduces the LTD lobe of the STDP curve, as show in Figure 3A, and provides a principled explanation for it. While the SF does indeed reproduce the general *shape* of the LTD lobe, the STDP protocol using the SF seems to have a vertical shift, in which long pre-before-post or post-before-pre delays result in a reduction in the synaptic weight, unlike what I've seen in standard STDP protocols, where long delays do not result in a weight change. Can the authors explain this discrepancy between standard STDP results and those produced by the SF?

Indeed, our theory predicts a negative vertical shift while standard results on STDP typically don't report a negative bias (Bi and Poo 1998; Froemke et al. 2005; Feldman 2000). There are however several points to note.

First, it should be mentioned that all the STDP results are very noisy so that STDP is typically described with simple models such as an exponential decay for the LTP and another one for the LTD. By actually looking closer to experimental data, it is not unreasonable to think that STDP results could be equally described (or even better described) with an additional negative bias (see Fig 1d in (Froemke et al. 2005) and Fig 5B in (Feldman 2000)).

Secondly, this vertical shift is also present in other normative models of STDP, (Pfister et al. 2006; Brea et al. 2013). It reflects the fact that if there is only a presynaptic spike - that does not lead to any postsynaptic spike, then in order to make the non-spiking of the postsynaptic neuron as likely as possible, this connection should be depressed.

Finally, in our model, this vertical shift (and actually the whole LTD lobe) is tuned by the parameter g_0, so if g_0 is small, then this vertical shift becomes negligible.

(4) One of my major issues with the paper was that I don't think the authors have remotely established neural plausibility. First, the dimensionalities used, < 20, seem to be significantly lower than those relevant to most tasks real neural systems would be solving, even accounting for sparsity. Perhaps the authors can be more specific about the setting in which they think their rule would apply? The authors suggest some ways to address this at the end of section 2.2, but I think, given the importance of these issues to biological plausibility, the suggestions should be fleshed out and incorporated into the paper. Also, the performance of the model in cases of model mismatch seems to drop rapidly with dimensionality, as shown in Figure 2E. Since it's likely that the brain is operating under model mismatch, it is alarming to see the performance of the SF drop so precipitously with the level of mismatch. The authors counter that the performance is relative to that of the gradient-based rule with optimized learning rates, but it would seem that optimizing a single learning rate can be done much more plausibly than tracking a full covariance matrix. This brings me to my second issue regarding neural plausibility: the SF is indeed normative, but requires the tracking of covariances between synaptic weights. How do the authors imagine this could possibly done in a realistic biological setting? The authors

are aware of this problem and mention some possible solutions in the discussion, but I would want these to be pursued in the main text, because without some hope of neural plausibility, I don't see how the SF, despite its normative benefits, is neuroscientifically relevant. This problem is particularly acute because the authors, to their credit, have investigated the performance of the diagonal synaptic filter, which would be more plausible. Unfortunately, as the authors show, its performance is often significantly worse than that of the SF, and also cannot explain heterosynaptic plasticity. Thus it seems that tracking of non-diagonal covariances is crucial, but it seems entirely unclear how it would be done in a biologically plausible setting.

We would like to thank the reviewer for this very relevant comment. Indeed the neural plausibility question needs to be better addressed since it is unclear how d synapses could represent and update the $d \times d$ posterior covariance matrix. In order to address this issue, we derived a Block Synaptic Filter (bSF) which is much simpler than the Full Synaptic Filter (fSF) and therefore more biologically plausible. The Block Synaptic Filter is a projection filter on the space of multi-variate Gaussian densities with block-diagonal covariance matrices. The bSF therefore interpolates between the Diagonal Synaptic Filter (dSF) and the Full Synaptic Filter. Because of the block diagonal structure of the bSF, the number of variables to track for the posterior covariance matrix scales as $d \cdot (b+1)/2$ where b is the size of the block. So the bSF is still linear in d if b is kept constant - which is much better than the quadratic scaling of the fSF.

Practically, the bSF assumes that each synapse can have access to information from nearby synapses (i.e. from all the synapses that belong to the same block) but don't need to be informed about the state of other synapses. This assumption of blocks of synapses could be implemented by the group of synapses that target the same dendritic branch. Indeed, it has been shown that synapses targeting the same dendritic branch typically receive correlated inputs while the inputs associated to synapses that belong to separate branches are uncorrelated (Takahashi et al. 2012).

So we adapted the manuscript in several places. We added in the Supplementary information the derivations of the bSF (Section C.5). We also substantially updated Fig 2 and updated the Results and Discussion sections.

Here we want to stress the fact that bSF is one amongst many possible approximate filters. It has however the clear advantage of being derived from a principled perspective, i.e. from a projection filtering perspective. Our proposed bSF clearly opens the door to biological implementability while keeping the interesting heterosynaptic properties.

(5) Given the biological plausibility issues above, I also felt that the paper did not adequately test the SF against other, non-normative, models that *are* biologically plausible. For example, I believe the LTD lobe of STDP can be produced within the author's own framework by holding the variance constant at the identity and just updating the means. The authors themselves cite existing work in the literature outside of the normative that can also reproduce this lobe. Holding the variance constant is a (limited) example of the diagonal synaptic filter, which the authors show cannot reproduce heterosynaptic plasticity. But perhaps this latter effect can be explained by applying sparsity inducing priors to the weights; intuitively it would seem that such a prior would also induce heterosynaptic plasticity, but have a more plausible biological implementation. Of course, the advantage of the SF is not just that it reproduces certain observed effects, but it does so in a principled way. Nevertheless, given how hard it seems to be to implement the normative solution in a biologically plausible way, I think it's important to rigorously test the SF against non-normative alternatives, as these alternatives might reflect the biologically plausible solutions that the brain has developed to solve the problems the SF addresses, but in realistic settings.

In the previous version of the manuscript, it was indeed very unclear how to biologically implement the Synaptic Filter such that a comparison with more biologically plausible rules was much needed.

However, now that we have derived a novel learning rule, i.e. the block-diagonal Synaptic Filter (bSF) whose complexity scales linearly instead of quadratically as a function of the input dimension, we believe that we are now in a different situation. This bSF is much more biologically plausible (see also our response the reviewer's point).

At this stage, we want to insist on the fact that a normative model is conceptually very different from a biophysical model. The main purpose of a normative learning rule is not to propose

a biophysical implementation of the rule but to provide a normative explanation of observed phenomena and to make novel predictions. Proposing a truly biophysical model (where every variable has a believable biophysical labelling) that captures STDP as well as heterosynaptic plasticity would be very interesting but is clearly beyond the scope of the present paper.

As suggested by the reviewer, we now thoroughly compare the different versions of the Synaptic Filter with the gradient descent rule (GD) which precisely corresponds to the Diagonal Synaptic Filter with a fixed variance (see new Fig. 2D-E).

Note also that a maximum-a-posteriori learning rule (MAP rule) which corresponds to the gradient of the loglikelihood plus the gradient of the log (sparsity) prior wouldn't lead to the heterosynaptic properties that we described here. Indeed, the log-prior term would simply add a weight decay term while the heterosynaptic effects in our learning rule come from the covariance matrix Σ that multiplies the Hebbian pre-post term. So an LTP event in one synapse leads to LTD to a correlated synapse for our learning rule while we don't think this would happen in the case of a MAP rule with a sparsity inducing prior.

Finally, we would like to acknowledge the fact that our model is not the only one that does predict heterosynaptic plasticity. For example, the work of (Kreutzer, Petrovici, and Senn 2020) precisely showed that when performing natural gradient, the learning rule has to consider also non-local effects.

(6) Thus in summary, I like (1) the normative framework the SF provides, (2) its explanation for heterosynaptic plasticity, (3) that it, at least partially, captures the negative lobe of the STDP curve, and (4) that it makes testable predictions about weight change variance in STDP experiments, and I think the authors have the core of a strong manuscript here. However, I think they need to do significantly more work to establish the advantages and limitations of the SF in neurally plausible settings, such as rigorous testing of their SF in more realistic settings with larger dimensionality and with more and different types of model mismatch, and a broader comparison to the best available non-normative competitors. Only then will I be able to accept that the Synaptic Filter provides, in the words of the authors, "a serious candidate for a computational principle underlying plasticity."

As requested by the reviewer, we made a great effort to test the Synaptic Filter for larger dimensions (see new Figs. 2D and 2E) and to compare it with other learning rules.

In particular, we derived a more biological learning rule (the Block Synaptic Filter, bSF) from first principles and systematically compared this bSF as well as the Diagonal Synaptic Filter and the gradient rule against the Full Synaptic Filter (see new Figs 2C, 2D and 2E).

This major revision gave rise to a significant change of focus of the paper. In particular, the performance evaluation section (i.e. Section 2.2) contains now the more biological filter (i.e. the bSF) and is now focused on an extensive comparison of the MSE performance between the different filters (dSF, bSF and fSF) especially in high dimensions.

We decided to keep the focus on MSE and removed the old figures 2D and 2E which were concerned with another metric (i.e. the marginal likelihood in old Fig. 2D) as well as the implications of the model mismatch (old Fig. 2E). Indeed, if we want to introduce a new performance metrics (such as the marginal likelihood) there are a lot of degrees of freedom to study systematically (dimensionality, the degree of mismatch, the statistics of the input, etc…). Those interesting questions would significantly lengthen the already pretty long manuscript and may distract the reader from the main message of the paper. We therefore believe that studying other metrics would belong to a separate study.

(7) On a non-scientific note, I also want to mention that I found the presentation of the paper to be quite poor. Because the technical content of the paper was correct (I manually checked most of the equations) I did not let the poor presentation of the paper affect my decision. Nevertheless, the problems below suggest a lack of both attention to detail and respect for the reviewers' time and need to be addressed before any resubmission.

The figures: These were provided unlabeled and appended in low resolution to the end of the paper as figS3, figS5, figS4, figS1, figS2, fig3, fig2, fig1, fig4. The proper labels could only be determined from the hyperlinks to the corresponding high-res sources provided in the corner of each figure. Next, the figures were provided in the sequence above, i.e. out of order. Further,

the figures were mislabelled: figures S1-S5 are referred to as figures 5-9 in the main text. I'm not sure how this happened, whether on the journal's end or the authors, but it has not happened on previous papers I have reviewed for this journal. I had to spend a significant amount of time before I could even start the review to collect and assemble the figures in the correct order. Providing figures in this format suggests a lack of respect for my time as a reviewer. In the future, please ensure that high-quality vector graphics figures with the proper labels are provided in the correct order.

We feel really sorry for the poor quality of the presentation of the first version of the manuscript. We don't know neither why the compiler on the PlosCB website produced such a bad result. In order to be sure to have a properly readable manuscript, we have uploaded an additional fully compiled version of the manuscript where all the figures are correctly placed within the text.

(8) The text: First, the citation format used in the paper is bulky and inconsistent, frequently appending the last names of all authors to the citation. For example, on line 166, the paper by Liakoni et al. 2019 is referred to as "Liakoni et al. 2019 Liakoni, Modirschanechi, Gerstner, Brea". This is obviously not the desired format for the citation (why list Liakoni twice, why use 'et al.' but then list all the authors anyway), and the error is obvious with even a cursory glance at the paper. Secondly, the paper has numerous minor errors. Most, but not all, were errors in spelling or grammar. In most cases these did not detract from the understandability of the content, but did from its readability. I've listed the ones I could find at the end of this review. Some of these errors weren't even buried in the text, but in section headings. Please ensure that the manuscript is properly proof-read before any future resubmission.

I thank the authors again for their submission.

We corrected the erroneous citation style.

3.3 Minor

- Abstract: "a better generalization performance" should be "better generalization performance"
- Abstract: "consistent with the STDP" should be "consistent with STDP"
- 106: No comma after "generative model"
- 199: For all four filtering models .... as depicted in Figure 2B: Only two models are shown in that panel, not four.
- 237: "in presence" should be "in the presence"
- 261: Synpatic should be Synaptic.
- 275: "differences in model evidence can be more directly attributed". More directly than what? Presumably MSE? But the sentence before just said that MSE is not even defined when there's model mismatch. So get rid of 'more' and just say 'can be directly attributed'
- 283: "less dimensions" should be "fewer dimensions"
- 304: Missing 'with': Should be "The Synaptic Filter is consistent with STDP"
- 319: "Matter debate" should be "Matter of debate"
- 338: "experiment" should be "experiments".
- 354: "is consistent" should be "are consistent."
- 373: "inputs spikes" should be "input spikes"
- 467: "induction mechanism. In the case" should be "induction mechanism, in the case"
- 473: "stimulated" should be "simulated"
- 495: "Parametrising" should be "Parameterising"

- In section 4.1.2, on the second line $D_t := x_t, y_t$: The t subscripts on x and y should be taus.
- Equation 8 in that section: Some mention should be made that the proportionality there is only approximate, because x_t^ϵ is being used not x_t.
- Two lines after equation 8: Bernulli should be 'Bernoulli'
- 661: The identity matrix is typically blackboard I, not 1.
- 4.3.1, equation 16: The equality should be replaced by approximate equality, because it holds when $\tau_{ou} \gg tau_m$. Similarly in equation 17. Alternatively, the authors can just state somewhere that they're going to use equality to mean approximate equality in these cases.
- Equation 17: The quantity is the variance of u_t, not u_t^2.
- Further down, fourth line after equation 19, the variance is that of u_t, not u_t^2.
- Equation 20: Given Equation 19, the expression for c in Equation 20 is missing a β_0 in the denominator.
- Equation 21: The statement before the equation is "Assuming a flat model prior, we have", but then what's written in the equation is the log *likelihood* of the model, which is what it is regardless of the prior. I think what's mean is that in the case of a flat model prior, the log model evidence is equal to the log likelihood up to constant term. So perhaps the equation should be written in terms of the log evidence as $\log p(M|D_t) = ... + constant$.
- 1064: "and it cannot influence" should be "and cannot influence"
- 1130: asymtotically should be asymptotically
- 1133: "higher dimension" should be "higher dimensions"
- Caption for Fig 6 near line 1151: "all filtering distribution" should be "all filtering distributions"; "particle filer" should be "particle filter"
- Line after eqn. 30: "dependent" should be "depend"
- Eqn 44: The second equation is for $d\Sigma_t$, not $d\Sigma_t^2$
- Line immediately before Eqn. 57: dtdelta should be ddelta.
- Eqn 57: This equation is unclear; first, what does the proportionality mean? Eqn 44 is exact, and all that's happening here is a substitution. Secondly, the terms proportional to dt in Eqn 44 are missing here; they reappear in Eqn. 59. If the intention is to discuss only the terms proportional to ddelta nd dN here, then a different symbol should be used. It's probably easiest and clearest to just write down what dSigma is after making all the substitutions, rather than leave parts out.
- 1244: "predictive of the gradient" should be "predictive performance of the gradient"
- 1254: "selected its maximum value of the loglikelihood" should say "selected the maximum value of the fit to the loglikelihoods."
- 1260: "to maximum value" should be "to the maximum value"
- 1280: "negative lobe dependence" should be "negative lobe depends"
- 1281: "do not" should be "does not"
- Caption to Fig. 8 (following line 1295): "The returns to its" should be "The mean value of the bias returns"
- The authors mention Kushner 1964 as an early example of filtering in the literature, but what about Norbert Wiener's 1949 "Extrapolation, Interpolation, and Smoothing of Stationary Time Series"?

We thank the reviewer for his detailed reading. We corrected all those minor comments.

References

Bi, G.-Q. and M. Poo (1998). Synaptic modifications in cultured hippocampal neurons: dependence on spike timing, synaptic strength, and postsynaptic cell type. Journal of Neuroscience 18(24), 10464–10472.

Brea, J., W. Senn, and J.-P. Pfister (2013). Matching Recall and Storage in Sequence Learning with Spiking Neural Networks. Journal of Neuroscience 33(23), 9565–9575.

Eden, U. T., L. M. Frank, R. Barbieri, V. Solo, and E. N. Brown (2004). Dynamic analysis of neural encoding by point process adaptive filtering. Neural Computation 16(5), 971–998.

Feldman, D. E. (2000). Timing-based LTP and LTD at vertical inputs to layer II/III pyramidal cells in rat barrel cortex. Neuron 27(1), 45–56.

Frey, U. and R. G. Morris (1997). Synaptic tagging and long-term potentiation. Nature 385(6616), 533–536.

Froemke, R. C., M.-m. Poo, and Y. Dan (2005). Spike-timing-dependent synaptic plasticity depends on dendritic location. Nature 434(7030), 221–225.

Jolivet, R., A. Rauch, H.-R. Lüscher, and W. Gerstner (2006). Predicting spike timing of neocortical pyramidal neurons by simple threshold models. Journal of Computational Neuroscience 21(1), 35–49.

Kreutzer, E., M. A. Petrovici, and W. Senn (2020). Natural gradient learning for spiking neurons. In Proceedings of the Neuro-inspired Computational Elements Workshop, pp. 1–3.

Krogh, A. and J. A. Hertz (1992). A simple weight decay can improve generalization. In Advances in neural information processing systems, pp. 950–957.

Pfister, J.-P., P. Dayan, and M. Lengyel (2010). Synapses with short-term plasticity are optimal estimators of presynaptic membrane potentials. Nature Neuroscience 13(10), 1271–1275.

Pfister, J.-P., T. Toyoizumi, D. Barber, and W. Gerstner (2006). Optimal spike-timing-dependent plasticity for precise action potential firing in supervised learning. Neural Computation 18(6), 1318–1348.

Surace, S. C. and J.-P. Pfister (2019). Online Maximum-Likelihood Estimation of the Parameters of Partially Observed Diffusion Processes. IEEE Transactions on Automatic Control 64(7), 2814–2829.

Takahashi, N., K. Kitamura, N. Matsuo, M. Mayford, M. Kano, N. Matsuki, and Y. Ikegaya (2012). Locally Synchronized Synaptic Inputs. Science 335(6066), 353–356.
