# Supplementary material for: Learning as filtering: Implications for spike-based plasticity
Source: PLoS Comput Biol. 2022 Feb 23;18(2):e1009721. doi: 10.1371/journal.pcbi.1009721 (PMC8865661; doi:10.1371/journal.pcbi.1009721)
Supplement: Attachment — Submitted filename: report2.pdf [file pcbi.1009721.s003.pdf]

The authors did a lot of work for this iteration of the manuscript, and I think it did improve as a result.

However, my main point of concern still remains. Namely, I still think that the claim to the effect that "[...] the performance simulations show that the Synaptic Filters substantially outperform a gradient rule with an optimal learning rate" (line 248, and in other forms at other places) is not properly substantiated, for the following reasons:

1. *The performance metrics are not the same for the synaptic filter (SF) and gradient descent (GD)*
   For the SF, the mean squared error (MSE) involves the teacher weights and the expected weights of the SF. For GD, since there is no explicit reference to the mean weights, its MSE (which is not defined in the manuscript) must involve the teacher weights and the (student) weights themselves. This means that the MSE for SF, as defined by the authors, systematically underestimate or misrepresent the actual MSE, namely the one which accounts for the randomness of the student weights. Indeed, we have that $\langle (w_t^* - w_t)^2 \rangle = \langle (w_t^* - \mu_t)^2 \rangle + \langle (\mu_t - w_t)^2 \rangle$, where w^* denote the ground truth (teacher) weights (not related to w^* in Fig.1), w the stochastic weights of the student model, and μ their mean. In other words,

   $$\mathrm{MSE}_{\mathrm{GD}} = \mathrm{MSE}_{\mathrm{SF}} + \langle (\mu_t - w_t)^2 \rangle.$$

   For instance, in the case where the determinism parameter $\beta = 0$, for GD w goes to zero so that $\mathrm{MSE}_{\mathrm{GD}} = \sigma_{\mathrm{OU}}^2$; for SF, according to Eqs. 5-6, using $\mathrm{MSE}_{\mathrm{GD}}$ instead of $\mathrm{MSE}_{\mathrm{SF}}$ would give $\mathrm{MSE}_{\mathrm{GD}} = 2\sigma_{\mathrm{OU}}^2$, namely twice the $\mathrm{MSE}_{\mathrm{GD}}$ of GD. Therefore, I think that using $\mathrm{MSE}_{\mathrm{GD}}$ for both GD and SF in Fig. 2A would yield different results.

   Note that I fully understand that SF provides inferences whereas GD provides point estimates via optimization; but at any point in time a neuron is dealing with a definite value for the weights. For a given sample realization of the Assumed Density Filter, you still produce a single weight vector at each time instant, which produces a given $\mathrm{MSE}_{\mathrm{GD}}$; different samples produce different $\mathrm{MSE}_{\mathrm{GD}}$. Technically, one could produce n random samples of the SF, compute the $\mathrm{MSE}_{\mathrm{GD}}$ for each of them, and then see whether the sample mean of $\mathrm{MSE}_{\mathrm{GD}}$ for the SF is significantly smaller that the $\mathrm{MSE}_{\mathrm{GD}}$ for GD. In other words, that would mean taking into account the contribution of the "bands" in Fig. 1D to the performance of the SF.

2. *Inference and optimization are not performed with the same constraints.*
   I've already mentioned part of this problem in my previous review, namely when mentioning that the SF has insider information about the teacher via the parameters μ_{OU}, σ_{OU} and τ_{OU}. I did not think the rebuttal was very convincing on this point. Even though GD optimizes the learning rate, it is unclear how this compares to having access to hidden model parameters. One could argue that knowledge of aspects of the "hidden" weight dynamics might instead favor SF over GD. Thus, being very assertive about the model performance comparison seems premature. In summary, SF performs inference using (very sloppily) $p(w|\text{Data}, \sigma_{\text{OU}}, \tau_{\text{OU}}, \mu_{\text{OU}})$ whereas GD optimizes $p(w|\text{Data})$ with hyperparameter tunings. A complete inference would require the generative process to be properly hidden, i.e. inferring σ_{OU}, τ_{OU}, μ_{OU}.

3. *The number of parameters are different for the two approaches.*
   GD has d free parameters to descend on, plus the learning rate hyperparameter. fSF (full SF) has $d(d+3)/2$ free parameters and at the other extreme dSF has $2d$ parameters. This mismatch in the number of parameters could bias the performance comparison on the training set, if the comparison was made with the same constraints and with the same performance metric.

Although I think the claim that the SF outperforms GD is unsupported, the paper is still interesting since the SF provides a nice insight into synaptic plasticity from the Bayesian perspective.

Other comments

- In trying to reproduce your results from Fig. 2A by myself, I noticed that the parameters for it are not clearly listed in the Methods (the methods seemingly start with Fig. 2B). Also, what were the initial conditions for μ and Σ in Eqs. 5-6? Initializations are mentioned at lines 643-645 but were these initializations specific to section 2.3-2.5 (as the leading paragraph of section 4.4 suggests)? What about the initialization for section 2.2? I also had some trouble maintaining the positive-definiteness of the SF covariance matrix, but perhaps my implementation is to blame.

- The value of τ_m should appear at some point in the main text or the methods (I didn't see it).

- I think it would help future readers to discuss how this contribution differs from the recently published paper Aitchison...Latham, Nat Neurosci 2021 in the intro.

Section-specific comments

(Note: LXXX stands for line XXX.)

Introduction, L65-66 References missing for the claim that "Normative models ... view of the pre-post LTP lobe." You may use the references from L271.

Section 2.1, in unnumbered paragraph before Eqs. 2-3 "we assume a stochastic leaky-integrate-and-fire neuron, also called Spike-Response Model". The Spike-Response model and the LIF model are not synonyms. The LIF model can be cast as a SRM, as done in Gerstner's book, but not all SRM models can be cast as a pure LIF. Saying "we assume a spike-response model" would be sufficient.

P. 5 footnote 2 This definition should be in the main text before Eq. 4. Or at least not in a footnote.

Section 2.2, L190 "both filtering models" → all filtering models

Section 2.2, L228 Unless I'm mistaken, the claim about the performance increase with τ_{OU} is not substantiated.

Section 2.2, L233 Homogeneous and constant input rates, on the one hand, and sparsity, on the other hand, are not mutually exclusive. Here, you actually want to justify the block-wise inputs by the desire to have sparse inputs without having to scale β.

Fig. 2B "determinsm" → determinism, and there should not be an hyphen in "mean squared error".

Fig. 3 I suggest to put panel C first, since it is mentioned first in the results. I also suggest to use the blank space in the figure canvas to illustrate the protocol.

Section 2.4, L334 I did not understand "First, it affects the membrane potential with this additional source of stochasticity (the sampled weight) and therefore impacts the form of the Synaptic Filter.", especially "the sampled weight" in parentheses. Aren't these weight sampling already present in the SF? Why would you need to amend the theory? The reason for introducing the Sampling SF is unclear.

Section 2.4, L337 The "Stochastic Synaptic Filter" is called the "Sampling Synaptic Filter", L882 in the SI.

Section 2.4, L351 "temporally": Did you mean "temporarily"?

Section 2.4, L353 "temporally": Did you mean "temporary"?

Section 2.5, L363 Remove comma between "homosynaptic" and "Hebbian".

Section 2.5, L368 "competes" → computes?

Section 2.5, L433 You would have other sources of variability in the model if you used the weights themselves and not their expectations. You would have a distribution orthogonal to the line in Fig. 4E.

Section 4.3, L604 Remove the second “terms”.
