# Supplementary material for: Learning as filtering: Implications for spike-based plasticity
Source: PLoS Comput Biol. 2022 Feb 23;18(2):e1009721. doi: 10.1371/journal.pcbi.1009721 (PMC8865661; doi:10.1371/journal.pcbi.1009721)
Supplement: Attachment — Submitted filename: 21-11-30-plos-cb-response-to-reviewers.pdf [file pcbi.1009721.s004.pdf]

Response to Reviewers for the manuscript "Learning as filtering: implications for spike-based plasticity"

Jannes Jegminat, Simone Carlo Surace and Jean-Pascal Pfister

Version: November 30, 2021

1 Reviewer 2

1.1 Major

I thank the authors for their submission of the revised manuscript, (and for including the fully compiled manuscript, which made reading the submission much easier). I appreciated the focus on characterization using MSE, and the extended characterization of performance with respect to dimension. As in the original submission, I also very much appreciate the normative approach to synaptic plasticity proposed here. However, as I'm sure the authors will agree, the normative approach is most illuminating when the resulting derivation is biologically plausible. I don't think the authors have established plausibility, as I describe below.

First, the strengths: I believe these are:

- The normative derivation, and the 'learning as filtering' approach.
- The explanation of the negative lobe of STDP
- The explanation of heterosynaptic plasticity
- The prediction about spike-timing-dependent changes in EPSP variance.
- The elegant derivation of the Block Synaptic Filter by projecting the covariance dynamics of the full synaptic filter into the space of symmetric, block-diagonal matrices; also the potential biological implementation of this filter by synapses on dendritic branches.
- The discovery of one regime of intermediate signal dimensionality where the block and full synaptic filters clearly outperform the diagonal synaptic filter (Fig. 2E).

The main weakness of the model remains its biological plausibility. The original model (the Full Synaptic Filter) was implausible for two reasons: (1) it requires the storage of $O(d^2)$ covariances for d synapses, and (2) these values, as covariances, are specific to pairs of synapses. The authors have addressed the first problem by deriving the Block Synaptic Filter, which requires fewer values to be stored, a number which importantly is now linear in d instead of quadratic. However, they have not addressed (nor even acknowledged) the second problem of how it would be possible not only for pairs of synapses, even those on the same dendritic branch, to store and update pair-specific covariance values. This might be possible if the synapses are immediately adjacent – corresponding to a tridiagonal structure of the covariance matrix – but otherwise it's difficult for me to see how non-adjacent synapses on a branch could coordinate each of their pairwise covariances independently. I want to emphasize that I'm not asking for biophysical plausibility (nor was I in my original review) – but the less strict 'biological plausibility': whether it is at all possible for synapses on the same branch that are not immediately adjacent to share and update pair-specific quantities.

Because synaptic machinery is complex and is still being explored, the apparent implausibility may perhaps be rendered plausible by some as yet unknown (at least to me) inter-synaptic

mechanism. Therefore, I would ask that the authors at least flesh out in some detail what the biological requirements for their update rules are, in particular the requirement implied by Eqn 6 for some form of communication between synapses to store the covariances. Additionally, equation 5 seems to require that even the mean value of each synapse is a linear weighting of the input at all synapses x_t by the appropriate elements of the synaptic covariance matrix. This again would seem very difficult to implement given what's known (at least to me) about synaptic and inter-synaptic machinery. Again, I'm not asking for full biophysical details, but just spelling out who needs to communicate what with whom.

On a lesser note, the authors show only one regime where the block and full synaptic filters significantly outperform the (much more plausible) diagonal synaptic filter (Figure 2E). This was in the setting in which the input had the block-diagonal covariance structure suitable for the Block Synaptic Filter. Even though the covariance in the model is meant to capture both environemental dynamics and parameter uncertainty, I wondered why the authors didn't also try a setting in which the weight dynamics (Eqn 1) included correlations? This might have allowed the three models to be better distinguished.

In summary I appreciate the normative derivation and the additional exploration of the performance of the Synaptic Filter but it still remains biologically implausible given current knowledge (at least mine) of synaptic and dendritic mechanisms, so I ask that the authors at least specify in some detail what the biological requirements for their model are.

We thank the reviewer for insisting on the biological plausibility question. We address this point by replacing the paragraph on biological plausibility in the Discussion with a more precise paragraph:

"Is the SF biological plausible?

First, it should be recalled that the Synaptic Filter (and all its variations) is a normative learning rule derived from some computational principle and does not predict in itself how the implementation should be. Indeed, in Marr's 3 level perspective, a computational principle can be achieved by many algorithm and every algorithm can be implemented in different ways, so the bottom-line is that the implementation is not unique.

If the implementation of the learning rule follows exactly the Eqs (5) and (6), it is indeed hard to see how this is biologically plausible. Taken as such every synapse i depends on the activities of all other inputs in a non-trivial way which seems to violate any sense of locality - which would be desirable for a biologically plausible learning rule. It is however possible to have other implementations that are consistent with Eqs (5) and (6) (or at least closely approximate those equations) and yet have a higher degree of biological plausibility. This would be for example the case for the constant Synaptic Filter which assumes that the inputs are roughly constant, i.e. $x^\epsilon \simeq \bar{x}$ (see Supplementary Information, Section F). In this case, we can define a surrogate variable $z = \Sigma\bar{x}$ such that synapse i depends only on z_i whose dynamics depends only on itself as well as a global factor $z^T\bar{x}$ which is available at all synapses. Further research is therefore required to determine which implementation best matches biophysical constraints while keeping the end-effects as predicted by Eqs (5) and (6)."

Regarding the correlations in the weight dynamics, we could have indeed introduced correlation in the weight dynamics either by having a non-diagonal drift or a non-diagonal diffusion. It should however be noted that many different prior weight correlations lead to the same spiking statistics (provided that the input spike train vector is adapted accordingly). Indeed, if we consider a weight vector w' which is related to the ground truth weight w by $w' = Aw$, where the matrix A is assumed to be invertible, the generative model can be re-expressed as

$$dw' = -\frac{w' - \mu'}{\tau_{\rm ou}}\mathrm{d}t + \sqrt{2\sigma_{\rm ou}^2\tau_{\rm ou}^{-1}}A dW \qquad (1)$$

$$y_t \sim \mathrm{PoissonProcess}(g(w'^T x_t'^\epsilon)) \qquad (2)$$

with $\mu' = A\mu$ and $x_t'^\epsilon = A^{-T}x_t^\epsilon$. So here the spiking statistics will be exactly the same as the one in the paper for any invertible A.

1.2 Minor

- The authors should explain better their differential-geometric derivation of the block-synaptic filter. For example, why is the procedure described on lines 1107-1110 the right thing to do? My guess is that the tangent space to the manifold of symmetric positive-definite matrices (i.e. covariance matrices) is isomorphic to the space of symmetric matrices Sym(d), and we want to project elements of this tangent space onto a block-diagonal subspace. Why is the Fisher-Rao metric the right metric with which to do this? We agree that the derivations of the block-diagonal filter was a bit compressed. We rewrote major parts of section C.5, better explaining how the derivations lead to the block-diagonal projection filter. Also, the argument about the isomorphism between the tangent space $T_\Sigma d$ and the the space of symmetric matrices d was present in the last version when we wrote $T_\Sigma d \cong d$, but was indeed succinct. So we now better explain the isomorphism. Finally, we added a footnote to justify the use of the Fisher-Rao metric.

- On line 224 the authors claim a particular difference in performance is 'significant'. Do they mean statistically significant? If so, please provide the statistics. If not, please clarify. We agree that 'significant' was a poor choice and changed the wording to 'consistent for $d < 128$' which more clearly makes the point that for a range of dimensions the order of the performance curves is the same, i.e., it is consistent.

- On line 233 the authors say: "contrary to the assumption of homogeneous, constant input rates ρ, neuronal activity in the brain is often sparse, i.e., most neurons are silent most of the time." But there is no contradiction between 'Most neurons being silent most of the time' 'homogeneous, constant input rates', only requires that these rates be low. We thank both reviewers for pointing out this inconsistency. It is indeed correct that the notion of "constant (Poisson) rate" is not necessarily inconsistent with the notion of "most neurons being silent most of the time". Indeed a low Poisson firing rate would satisfy those 2 conditions. We added the following paragraph to explain that the block-wise sparse inputs are motivated by our desire to match the learning rule's block structure to the inputs:

  "Studying the performance in high dimensions, we have made two assumptions: first, that the input firing rates are homogeneous and constant, and, secondly, that the average output firing rate of the neuron is kept constant via the scaling of determinism parameter β. Another way to get a consistent scaling with respect to the input dimensionality is to have a block-sparse structure in the input, referred to as *sparse* for short, i.e., at any time there are only b neurons active. More precisely, the input neurons are divided into blocks of size $b = 8$ and activated one block at a time for the duration of $\tau_{\text{block}} = 1$ s. This block-sparse input structure is a simple implementation of the fact that inputs that target the same dendritic branch are highly correlated (Kleindienst, Winnubst, Roth-Alpermann, Bonhoeffer, and Lohmann 2011). With one block active at any point in time, the expected output firing rate does not depend on the total number of blocks, i.e., the total dimensionality. Thus, in contrast to the previous simulations, there is no need to scale β to keep the output neuron's statistics invariant."

- Line 204,252: 'block' should be 'Block' Done
- Line 237: 'dependent' should be 'depend' Done
- Line 368: 'competes' should be 'computes' Done
- Line 501: 'don't' should be 'doesn't' Done
- Line 611: 'burnin' should be 'burn-in' Done
- Line 858: First quotation mark is backwards Done
- Line 925: 'burn in' should be 'burn-in' Done
- Line 1114: There's an extra tab before 'where' Done

We appreciate the reviewer's careful reading of the new version and fixed the aforementioned issues.

2 Reviewer 3

2.1 Major

The authors did a lot of work for this iteration of the manuscript, and I think it did improve as a result. However, my main point of concern still remains. Namely, I still think that the claim to the effect that "[...] the performance simulations show that the Synaptic Filters substantially outperform a gradient rule with an optimal learning rate" (line 248, and in other forms at other places) is not properly substantiated, for the following reasons:

1. The performance metrics are not the same for the synaptic filter (SF) and gradient descent (GD) For the SF, the mean squared error (MSE) involves the teacher weights and the expected weights of the SF. For GD, since there is no explicit reference to the mean weights, its MSE (which is not defined in the manuscript) must involve the teacher weights and the (student) weights themselves. This means that the MSE for SF, as defined by the authors, systematically underestimate or misrepresent the actual MSE, namely the one which accounts for the randomness of the student weights. Indeed, we have that $\langle (w_t^\star - w_t)^2 \rangle = \langle (w_t^\star - \mu_t)^2 \rangle + \langle (\mu_t - w_t)^2 \rangle$, where $w^\star$ denote the ground truth (teacher) weights (not related to $w^\star$ in Fig.1), w the stochastic weights of the student model, and μ their mean. In other words, $MSE_{GD} = MSE_{SF} + \langle (\mu_t - w_t)^2 \rangle$. For instance, in the case where the determinism parameter $\beta = 0$, for GD w goes to zero so that $MSE_{GD} = \sigma^2$; for SF, according to Eqs. 5-6, using MSE_{GD} instead of MSE_{SF} would give $MSE_{GD} = 2\sigma^2$, namely twice the MSE_{GD} of GD. Therefore, I think that using MSE_{GD} for both GD and SF in Fig. 2A would yield different results. Note that I fully understand that SF provides inferences whereas GD provides point estimates via optimization, but at any point in time a neuron is dealing with a definite value for the weights. For a given sample realization of the Assumed Density Filter, you still produce a single weight vector at each time instant, which produces a given MSE_{GD}, different samples produce different MSE_{GD}. Technically, one could produce n random samples of the SF, compute the MSE_{GD} for each of them, and then see whether the sample mean of MSE_{GD} for the SF is significantly smaller that the MSE_{GD} for GD. In other words, that would mean taking into account the contribution of the "bands" in Fig. 1D to the performance of the SF.
   We thank the reviewer as well as the editor for giving us a second chance to dissipate misunderstandings regarding the comparison between the Synaptic Filter (SF) and the gradient descent (GD) rule. Actually both the SF and the GD are evaluated in the very same way. Indeed for both rules, we compute the mean squared error between the teacher weight $w_t^\star$ and the best estimate from the given learning rule (i.e. $\hat{w}_t$ for GD and μ_t for SF). Therefore, for the case $\beta = 0$ and the initial conditions $\mu_0 = \hat{w}_0 = 0$, the MSE of the Synaptic Filter and the gradient rule corresponds the variance of the teacher's weight: $MSE = \sigma_{OU}^2$.
   We also made the definition of the MSE explicit for the Gradient Descent rule by adding another sentence. The first paragraph at the beginning of Section 2.2 now reads: "The MSE is defined by MSE $:= d^{-1} \langle (w_t - \mu_t)^\top (w_t - \mu_t) \rangle_t$ where w_t denotes the teacher's weight and μ_t is the best estimate of the Synaptic Filter. In the case of the gradient rule, μ_t is replaced with $\hat{w}_t$, the best estimate of the weight."

2. Inference and optimization are not performed with the same constraints. I have already mentioned part of this problem in my previous review, namely when mentioning that the SF has insider information about the teacher via the parameters μ_{OU}, σ_{OU} and τ_{OU}. I did not think the rebuttal was very convincing on this point. Even though GD optimizes the learning rate, it is unclear how this compares to having access to hidden model parameters. One could argue that knowledge of aspects of the "hidden" weight dynamics might instead favor SF over GD. Thus, being very assertive about the model performance comparison seems premature. In summary, SF performs inference using (very sloppily) $p(w|Data, \mu_{OU}, \sigma_{OU}, \tau_{OU})$ whereas GD optimizes $p(w|Data)$ with hyperparameter tunings. A complete inference would require the generative process to be properly hidden, i.e. inferring μ_{OU}, σ_{OU} and τ_{OU}.

First, let us clarify what counts as a valid comparison. In our context, an algorithm A is a candidate for comparison if it transforms input spikes $\mathbf{x_t}$ and output spikes y_t into a weight estimate $\hat{w}_t$. The comparison is valid if the candidate algorithms are compared on the same metric. The simplest possible candidate would be to set $\hat{w}_t = 0$ independently of $\mathbf{x}_t$ and y_t. Even though rather stupid, this algorithm remains valid (and is actually the best in terms of MSE in the limit of $\beta \to 0$). Both the gradient descent algorithm and the SF algorithm are also valid algorithms for the comparison. Now if a given algorithm outperforms another one, this is in principle for a good reason. Invoking those reasons to justify the unfairness of the comparison itself seems a curious step.

Secondly, as pointed by the reviewer, there are indeed some good reasons why the SF outperforms the GD algorithm, namely the fact that the SF has some "insider information" about the parameters that govern the dynamics of the true weights. This being said, the GD algorithm has also some advantages, and in particular the fact that the learning rate is optimised to minimise the MSE. There to know which one is the winner, a systematic comparison was needed and this is what we did in Figure 2.

Finally, another valid algorithm would be to consider the SF where the parameters are not known but have to be learned. This is certainly an interesting question, but due to the large study that needs to be done, we decided to leave this for future work.

3. The number of parameters are different for the two approaches. GD has d free parameters to descend on, plus the learning rate hyperparameter. fSF (full SF) has $d(d+3)/2$ free parameters and at the other extreme dSF has $2d$ parameters. This mismatch in the number of parameters could bias the performance comparison on the training set, if the comparison was made with the same constraints and with the same performance metric.

   We agree with he reviewer that for a regression task, fitting a model with more parameters and evaluating it on the training set biases the comparison towards the more complex model. Simply put, fitting a 10th order polynomial (with 11 parameters) on some data will provide a lower fitting error (on the training set) than a 2nd order polynomial (with 3 parameters). However, the analogy to regression (or polynomial fitting) with our situation differs in several respect.

   Firstly, unlike the polynomial fitting example, which aims to fit data points with two different sets of parameters (3 parameters vs 11 parameters), our study compares both the GD method as well as the SF method, which try to estimate the same number of parameters, i.e. $\hat{\mathbf{w}} = (\hat{w}_1, \ldots, \hat{w}_d)$.

   Secondly, the fact that the *SF* method has more internal variables (i.e. $d(d+3)/2$) to describe the parameters than the number of parameters (d) is a natural consequence of the Bayesian approach. Actually any Bayesian method which reformulates learning as inference will have more internal variables than parameters. This of course doesn't prevent the comparison between Bayesian and non-Bayesian methods.

   Finally, the very notion of training and testing doesn't apply here. Indeed, since the mapping from input spikes $\mathbf{x}_t$ to output spikes y_t changes continuously, there is no real distinction between training set and testing set to be made.

Although I think the claim that the SF outperforms GD is unsupported, the paper is still interesting since the SF provides a nice insight into synaptic plasticity from the Bayesian perspective.

2.2 Minor

1. In trying to reproduce your results from Fig. 2A by myself, I noticed that the parameters for it are not clearly listed in the Methods (the methods seemingly start with Fig. 2B). Also, what were the initial conditions for μ and Σ in Eqs. 5-6? Initializations are mentioned at lines 643-645 but were these initializations specific to section 2.3-2.5 (as the leading paragraph of section 4.4 suggests)? What about the initialization for section 2.2? I also had some trouble maintaining the positive- definiteness of the SF covariance matrix, but perhaps my implementation is to blame. We thank the reviewer for these helpful comments. Generally, the burn-in removes the dependence on these initial conditions, however, we fully

agree with the reviewer that they should be included for completeness. In Section 4.3., we added the sentence: "The initial conditions for all performance simulations were $\mu_0 = \mu_{\text{ou}}$ and $\Sigma_0 = \Sigma_{\text{ou}}$".

In our simulations, a sufficiently small time step successfully kept the covariance update from overshooting (which is usually the cause of a violation of the positive-definiteness requirement).

We now list the simulation details for Fig 2.A in Section B.3 of the Supplementary Information and added a pointer to Section 4.3 (Materials and Methods) in the Main Text. The reason for placing the details in the Supplementary Information is that the results in Fig 2.A were obtained from the same set of simulations shown in Section A and Section B of the Supplementary Information.

2. The value of τ_m should appear at some point in the main text or the methods (I didn't see it). We added the value in the first paragraph on pg. 5, where the generative model is introduced.

3. I think it would help future readers to discuss how this contribution differs from the recently published paper Aitchison...Latham, Nat Neurosci 2021 in the intro.
We agree with the reviewer that it is important to highlight our novel contributions with respect to Aitchison [2021] more clearly. We updated the relevant paragraph in the introduction to highlighting that we use a continuous-time setting and weight correlation, and this allowed us to obtain novel biological results.

   Previously, it read:
"In a continuous-time, spiking neuronal network, we derive the update rule for the synaptic weight distribution and call it the Synaptic Filter. The Synaptic Filter is computationally relevant because it outperforms a gradient rule with optimised learning rate parameter in a dynamic weight estimation task, confirming a previous result [48]. From the biological perspective, [...]".

   Now it reads:
"In a continuous-time, spiking neuronal network, we derive the update rule for the synaptic weight distribution and call it the Synaptic Filter. The Synaptic Filter is computationally relevant because it outperforms a gradient rule with optimised learning rate parameter in a dynamic weight estimation task, confirming a previous result obtained in a discrete-time setting and without including weight correlations [48]. Leveraging the continuous-time setting and weight correlation, the Synaptic Filter makes three experimental predictions. First [...]"

4. Introduction, L65-66 References missing for the claim that "Normative models ... view of the pre-post LTP lobe." You may use the references from L271. Done

5. Section 2.1, in unnumbered paragraph before Eqs. 2-3 "we assume a stochastic leaky-integrate-and-fire neuron, also called Spike-Response Model". The Spike-Response model and the LIF model are not synonyms. The LIF model can be cast as a SRM, as done in Gerstner's book, but not all SRM models can be cast as a pure LIF. Saying "we assume a spike-response model" would be sufficient. We thank the reviewer for the clarification and, as suggested, removed the words 'stochastic leaky-integrate-and-fire neuron, also called'.

6. P. 5 footnote 2 This definition should be in the main text before Eq. 4. Or at least not in a footnote. Done

7. Section 2.2, L190 "both filtering models" $\rightarrow$ all filtering models Done

8. Section 2.2, L228 Unless I'm mistaken, the claim about the performance increase with τ_{OU} is not substantiated. Indeed, we did not include the evidence for our claim that larger τ_{OU} decreases the MSE and decided to drop the claim for the sake of simplicity.

9. Section 2.2, L233 Homogeneous and constant input rates, on the one hand, and sparsity, on the other hand, are not mutually exclusive. Here, you actually want to justify the

block-wise inputs by the desire to have sparse inputs without having to scale β. See answer to 3rd minor comment of the other Reviewer.

10. Fig. 2B "determinsm" → determinism, and there should not be an hyphen in "mean squared error". Done
11. Fig. 3 I suggest to put panel C first, since it is mentioned first in the results. I also suggest to use the blank space in the figure canvas to illustrate the protocol. We thank the reviewer for the suggestion and updated the manuscript accordingly.
12. Section 2.4, L334 I did not understand "First, it affects the membrane potential with this additional source of stochasticity (the sampled weight) and therefore impacts the form of the Synaptic Filter.", especially "the sampled weight" in parentheses. Aren't these weight sampling already present in the SF? Why would you need to amend the theory? The reason for introducing the Sampling SF is unclear. We'd like to point out that the weights are only drawn stochastically in the case of the Sampling Synaptic Filter, not in the other Synaptic Filters. The Synaptic Filter encodes the weight uncertainty with the mean and covariance of the posterior and uses it in the update rules. However, the Synaptic Filter does not rely on the assumption that weights are sampled from the posterior. This additional assumption was only tested via the Sampling Synaptic Filter. To clarify this, we added the following sentence in the first paragraph of Section A of the SI: "However, the Synaptic Filter, as derived before, does not use weight sampling." The paragraph now reads:

    "The sampling hypothesis states that EPSPs are samples from the filtering distribution $p(w_t|\mathcal{D}_t)$ [59]. However, the Synaptic Filter, as derived before, does not use weight sampling. We wondered whether the sampling hypothesis could be used to inspire a version of the Synaptic Filter that maintains good filtering performance while at the same time computing the expected firing rate through EPSP samples."
13. Section 2.4, L337 The "Stochastic Synaptic Filter" is called the "Sampling Synaptic Filter", L882 in the SI. Done
14. Section 2.4, L351 "temporally": Did you mean "temporarily"? Done
15. Section 2.4, L353 "temporally": Did you mean "temporary"? Done
16. Section 2.5, L363 Remove comma between "homosynaptic" and "Hebbian". Done
17. Section 2.5, L368 "competes" → computes? Done
18. Section 2.5, L433 You would have other sources of variability in the model if you used the weights themselves and not their expectations. You would have a distribution orthogonal to the line in Fig. 4E. The Synaptic Filter is derived from the assumption that synapses have the capacity to represent correlations between weights in alignment with a Bayesian treatment of uncertainty. These high level assumptions (along with additional specifications of the model) are sufficient to recover the experimentally observed negative correlation between homo- and heterosynaptic plasticity during STDP experiments. As we know there are a lot of sources of variability in experimental data and therefore, we believe that predicting the extent of the orthogonal variability on this plot would be asking too much from the Synaptic Filter. Finally, including this additional filter in Fig. 4 might make the plot too busy. We also refer to our response to Minor Comment 12, i.e., the Synaptic Filter analysed in the Main Text does not sample from the weight distribution.
19. Section 4.3, L604 Remove the second "terms". Done

We thank the reviewer for the detailed reading. We corrected all those minor comments.

References

Kleindienst, T., J. Winnubst, C. Roth-Alpermann, T. Bonhoeffer, and C. Lohmann (2011). Activity-dependent clustering of functional synaptic inputs on developing hippocampal dendrites. Neuron 72(6), 1012–1024.
